# CANCERSIGN: a user-friendly and robust tool for identification and classification of mutational signatures and patterns in cancer genomes

**DOI:** 10.1038/s41598-020-58107-2

**Published:** 2020-01-28

**Authors:** Masroor Bayati, Hamid R. Rabiee, Mehrdad Mehrbod, Fatemeh Vafaee, Diako Ebrahimi, Alistair R. R. Forrest, Hamid Alinejad-Rokny

**Affiliations:** 1 Bioinformatics and Computational Biology Lab, Department of Computer Engineering, Sharif University of Technology, Tehran, 11365 Iran; 2 School of Biotechnology and Biomolecular Sciences, University of New South Wales, UNSW, Sydney, 2033 NSW Australia; 3 Quantitative Biology Lab, Texas Biomedical Research Institute, Texas, TX US; 4 Systems Biology and Health Data Analytics Lab, The Graduate School of Biomedical Engineering, UNSW Sydney, Sydney, NSW 2052 Australia; 5 School of Computer Science and Engineering, UNSW Sydney, Sydney, NSW 2052 Australia; 6 Harry Perkins Institute of Medical Research, QEII Medical Centre and Centre for Medical Research, The University of Western Australia, Nedlands, 6009 Australia

**Keywords:** Computational platforms and environments, Cancer genomics, Mutagenesis

## Abstract

Analysis of cancer mutational signatures have been instrumental in identification of responsible endogenous and exogenous molecular processes in cancer. The quantitative approach used to deconvolute mutational signatures is becoming an integral part of cancer research. Therefore, development of a stand-alone tool with a user-friendly interface for analysis of cancer mutational signatures is necessary. In this manuscript we introduce CANCERSIGN, which enables users to identify 3-mer and 5-mer mutational signatures within whole genome, whole exome or pooled samples. Additionally, this tool enables users to perform clustering on tumor samples based on the proportion of mutational signatures in each sample. Using CANCERSIGN, we analysed all the whole genome somatic mutation datasets profiled by the International Cancer Genome Consortium (ICGC) and identified a number of novel signatures. By examining signatures found in exonic and non-exonic regions of the genome using WGS and comparing this to signatures found in WES data we observe that WGS can identify additional non-exonic signatures that are enriched in the non-coding regions of the genome while the deeper sequencing of WES may help identify weak signatures that are otherwise missed in shallower WGS data.

## Introduction

Aberrant somatic changes in DNA resulting from endogenous sources (e.g. APOBEC-induced mutagenesis and DNA repair defects) and exogenous factors (e.g. tobacco smoking and UV radiation) are the hallmark of cancer. These alternations in DNA may have different forms, ranging from gross chromosomal rearrangements to single base substitutions^1^. The whole genome sequencing of tumor cells has shown that the number of mutations varies from less than one hundred per genome to hundreds of thousands depending on the cancer type and patient. Moreover, the type of mutation and sequence context of many cancer mutations are not random. For instance, C-to-T mutation within the CG (a.k.a. CpG) dinucleotide is a prevalent mutation in cancer and as its abundance is proportional to the age of patient it is referred to as an “aging” signature^2^. Many cancers also have a large number of C-to-T and C-to-G mutations within TCA and TCT trinucleotides^3^. These mutations are attributed to the aberrant changes in the level and activity of APOBEC enzymes. The mutational landscape of each cancer genome is thus a cumulative result of multiple mutational signatures, each caused by a unique process such as methylation, APOBEC mediated changes, etc.^1^.

Typically, signatures of mutational processes are determined by considering the trinucleotide context of single base substitutions. If all mutations are presented based on changes in the same DNA strand, there are 96 possible different types of mutations within trinucleotide motifs^4^. In 2013, Alexandrov *et al*. proposed a mathematical framework for analysing mutational signatures^4^ based on these 96 types of mutations. Using a matrix factorization algorithm, the authors uncovered 30 independent mutational signatures. They have recently updated the cancer mutational signature profiles by identifying 67 single base substitution mutational signatures^5^. Details of these signatures including their prevalence in each cancer type and potential etiology are available at the COSMIC database (http://cancer.sanger.ac.uk/cosmic/signatures ^6^).

The discovery of mutational signatures was a breakthrough in the field of cancer research. Therefore, the mathematical framework developed by Alexandrov *et al*.^4^ is now routinely used to identify novel mutational signatures and to study the processes involved in different cancers and in different patients. To help the progress of this field, we have developed a computational tool, CANCERSIGN, which enables the users to easily apply a matrix factorization analysis to cancer mutation datasets and receive a complete set of mutational signatures. Compared to the previously developed packages in R^7–9^, CANCERSIGN is unique in that it is a stand-alone package (i.e. it does not require additional software programming). Therefore, to use this tool, no programming skills are required. Additionally, it enables the users to perform *de novo* mutational signature analyses.

Application of CANCERSIGN is not limited to extracting mutational signatures based on nucleotides immediately flanking the mutated site (i.e. tri-nucleotide motifs). It allows the users to extend the analysis to two bases on each side of the mutated base (i.e. penta-nucleotides motifs). According to a recent study^10^, taking larger sequence contexts into consideration provides a greater power to explain variability in genomic substitution probabilities. In addition, CANCERSIGN allows the user to select trinucleotides of interest, and determine their penta-nucleotide mutational signatures. Furthermore, it has a built-in clustering option to study the groupings of cancer samples based on the raw mutation counts and/or composition of mutational signatures. In this manuscript, we introduce CANCERSIGN and show the new mutational signatures obtained from a *de novo* analysis of whole genome ICGC dataset. This analysis was performed for each cancer type separately and resulted in 77 mutational signatures. Each of the obtained signatures were shown to be highly similar to at least one of the 67 signatures discovered recently by Alexandrov *et al*.^5^, except two signatures that potentially can be considered as novel.

## Data and Methods

To develop CANCERSIGN and demonstrate its application, we used all the whole genome mutation datasets available at the International Cancer Genome Consortium (ICGC) data portal. A summary of the datasets used is shown in Supplementary Table S1.

**Figure 1:**
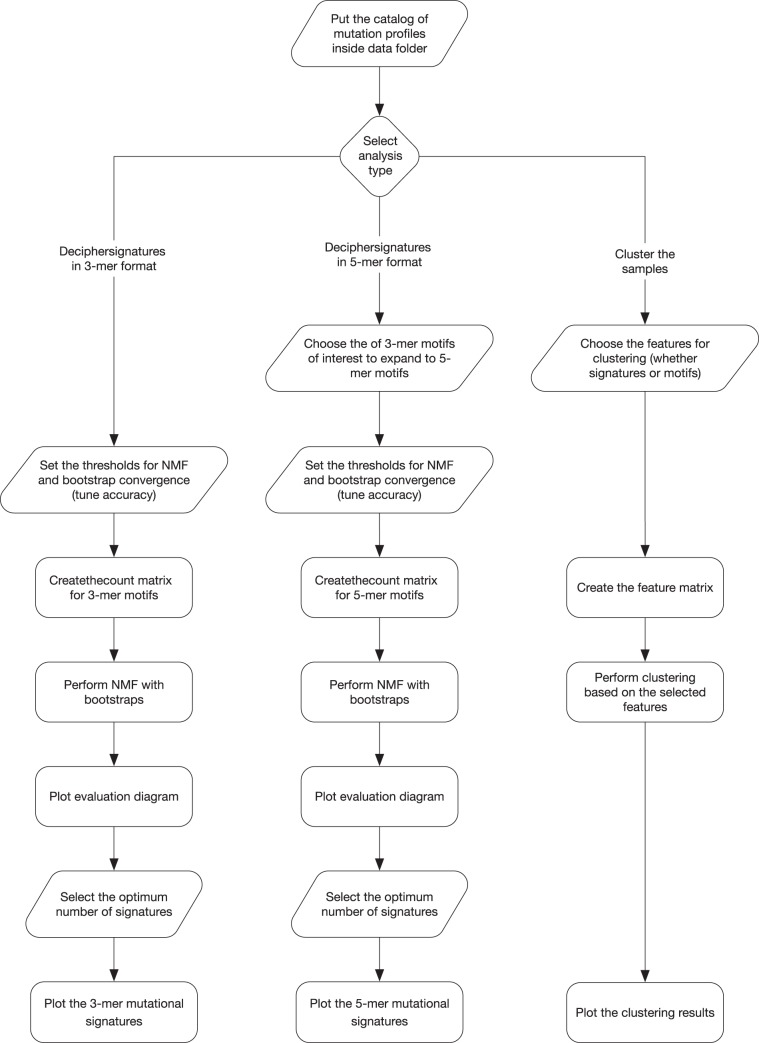
Tool functionality map. This diagram abstracts the functional features of CANCERSIGN. The user can either choose to extract mutational signatures or cluster the tumor samples. Signatures can either be deciphered in the conventional format which are the spectrums over 96 mutation types (3-mer motifs) or in 5-mer format. For extracting 5-mer signatures, the mutational motifs (penta-nucleotides) are determined based on the set of user defined 3-mer motifs and their flanking bases. In the clustering part, the user can choose different sets of features to cluster the samples. These feature sets are based on either counts of mutational motifs or based on the contribution of mutational signatures.

A schematic of CANCERSIGN features is given in Fig. 1. Basically, this tool deciphers mutational signatures in somatic mutation datasets using a previously reported non-negative matrix factorization (NMF) model^4^. It is also capable to cluster the tumor samples based on either the contribution of deciphered signatures to their mutational profiles or the mutational burden in 3-mers or 5-mers motifs chosen by the user. It only requires a dataset of mutations in a simple format described in the tool manual (https://github.com/bcb-sut/CANCERSIGN and https://github.com/forrest-lab/CANCERSIGN). Note that this tool performs its analyses based on the hg19 genome build.

CANCERSIGN then performs the requested analyses and outputs mutational signatures (3-mer and/or 5-mer formats) and clustering figures. The numerical outputs of the analyses are saved in a folder named “output” (refer to the CANCERSIGN manual). This data can be used to gain further insight into mutational processes in cancer. For example, the exposure matrix obtained from the NMF analysis, can be used to determine the prevalence of each mutational signature in each sample.

### Estimation of mutational signatures

Each mutational signature is defined by a total of 96 types of mutations within tri-nucleotide motifs^4^. Assuming that ${m}_{g}^{i}$ represents the number of mutations of type *i* recorded in the mutational catalogue of sample *g*, then the mutational catalogue of multiple samples is represented by a matrix *M* (*K* × *G*):

$$M=\left[\begin{array}{ccc}\begin{array}{cc}{m}_{1}^{1} & {m}_{2}^{1}\end{array} & \cdots  & \begin{array}{cc}{m}_{G-1}^{1} & {m}_{G}^{1}\end{array}\\ \begin{array}{cc}\,\vdots \, & \,\,\vdots \end{array} & \ddots  & \begin{array}{cc}\vdots \, & \,\,\vdots \end{array}\\ \begin{array}{cc}{m}_{1}^{K} & {m}_{2}^{K}\end{array} & \cdots  & \begin{array}{cc}{m}_{G-1}^{K} & {m}_{G}^{K}\end{array}\end{array}\right]$$

where, *K* represents the number of mutation types (*K* = 96 for tri-nucleotide signature analysis) and *G* represents the number of cancer samples. Therefore, the *i* th column of *M* has the number of each of 96 mutation types in the *i* th sample. Here, it is assumed that the mutational catalogue of each sample (i.e. each column of *M*) is the result of linear superposition of several mutational signatures^4^, each of which corresponds to a particular mutational process. Assuming that the number of mutational signatures is *N*, we need to factorize *M* into two matrices *P* and *E* with sizes *K* × *N* and *N* × *G*, respectively:

1 $$M\approx P\times E$$

The expanded representation of the above equation is given by:

$$\left[\begin{array}{ccccc}{m}_{1}^{1} & {m}_{2}^{1} & \cdots  & {m}_{G-1}^{1} & {m}_{G}^{1}\\ \vdots \, & \vdots  & \ddots  & \vdots  & \vdots \\ {m}_{1}^{K} & {m}_{2}^{K} & \cdots  & {m}_{G-1}^{K} & {m}_{G}^{K}\end{array}\right]\approx \left[\begin{array}{ccccc}{p}_{1}^{1} & {p}_{2}^{1} & \cdots  & {p}_{N-1}^{1} & {p}_{N}^{1}\\ \vdots \, & \vdots \, & \ddots  & \vdots \, & \vdots \,\\ {p}_{1}^{K} & {p}_{2}^{K} & \cdots  & {p}_{N-1}^{K} & {p}_{N}^{K}\end{array}\right]\times \left[\begin{array}{ccccc}{e}_{1}^{1} & {e}_{2}^{1} & \cdots  & {e}_{G-1}^{1} & {e}_{G}^{1}\\ \vdots \, & \vdots \, & \ddots  & \vdots \, & \vdots \,\\ {e}_{1}^{N} & {e}_{2}^{N} & \cdots  & {e}_{G-1}^{N} & {e}_{G}^{N}\end{array}\right]$$

Here, each column of *P* is interpreted as one mutational signature and each row of *E* represents the exposures of each mutational signature in the corresponding sample genome (i.e. the prevalence of each mutational signature in that sample). The process of estimating independent mutational signatures from a mutational catalogue matrix is done using a nonnegative matrix factorization (NMF) method. Note that the elements of the input catalogue matrix are nonnegative because they represent the number of mutations.

The algorithm used in this framework consists of several iterative steps. The overall procedure is as follows. Each iteration starts with sampling from matrix *M* and creating a new bootstrapped matrix $\breve{M}$. To do this, each column of *M* (mutation counts of one input cancer genome) is considered a discrete probability distribution and is resampled to create the corresponding column of $\breve{M}$. Next, the bootstrapped matrix $\breve{M}$ is factorized into matrices *P* and *E* by applying the NMF algorithm. These steps will be repeated for a certain number of iterations (typically 600 iterations are enough to obtain a stable result). The resulting matrices obtained after each iteration are stored and grouped into two sets: *P* matrices and *E* matrices named *S*_*P*_ and *S*_*E*_, respectively. The set of columns of matrices in *S*_*P*_ are then clustered into *N* groups using a variation of the $k-means$ clustering algorithm^11^. A similarity measure between two columns is calculated using cosine similarity and the centroids of the clusters are obtained by averaging over the members of each cluster. The cosine similarity between the vectors *A* and *B* is calculated as $A\cdot B/\sqrt{(A\cdot A)\times (B\cdot B),}$ where *A B* represents the dot product of vectors *A* and *B*. Finally, the *N* centroids are combined to form the columns of a single matrix $\bar{P}$, which is the matrix of mutational signatures. Since each column of matrix *P* corresponds to one specific row of matrix *E*, the procedure of clustering columns of matrices in ${S}_{P}$ naturally leads to clustering of rows of matrices in ${S}_{E}$. Therefore, the final exposure matrix $\bar{E}$ (prevalence of mutational signatures) is constructed in a manner similar to $\bar{P}$ after the clustering step. Finally, the results are evaluated by calculating the reproducibility of mutational signatures and the Frobenius reconstruction error of the NMF solution. The reproducibility is measured by calculating the average silhouette width of the result of clustering step, while the reconstruction error of the solution is quantified by the Frobenius norm of difference between matrix *M*, and its estimation obtained by the factorization algorithm, i.e., $M-\bar{P}\times {\bar{E}}_{F}^{2}$^4^. The overall procedure described above is carried out for different values of *N*; the number of deciphered signatures. The range of values for *N* can be specified by the user and the optimum value of *N* can be selected based on the aforementioned evaluation measures^4^. An optimum *N* leads to high reproducibility and low reconstruction error. CANCERSIGN produces an evaluation plot similar to the one proposed by Alexandrov *et al*.^4^.

CANCERSIGN is designed to carry out each iteration of the aforementioned algorithm, which consists of one bootstrap and a complete NMF decomposition, independent and in parallel with other iterations. This enables the user to distribute the whole task across CPU cores and speed up the procedure of extracting mutational signatures. This tool also allows the user to specify the convergence criteria, the number of NMF iterations, the number of bootstrapping iterations and the number of CPU cores for parallelization.

### Penta-nucleotide mutational signatures

In addition to extracting mutational signatures based on the immediately flanking nucleotides around the mutated site (tri-nucleotides), CANCERSIGN enables the user to further investigate the underlying mutational process, by expanding the mutational context to two bases upstream and downstream of the mutated base (penta-nucleotides). For this purpose, the user can choose an arbitrary set of mutations in tri-nucleotide motifs (up to 10 combinations). For each of these selected motifs, our tool quantifies the number of mutations in the corresponding 16 penta-nucleotide motifs (e.g. when the motif C[G > T]A is selected, 16 penta-nucleotide motifs of the form NC[G > T]AN are considered where N is each of four nucleotides). The mutation counts of penta-nucleotides in all samples are then used as the input to the NMF analysis, which produces a set of penta-nucleotide (or 5-mer) mutational signatures.

### Clustering of samples

One important application of CANCERSIGN is clustering of tumor samples based on the contribution of mutational signatures (Exposure Matrix *E* in Eq. 1) or based on the mutation counts within selected motifs (Matrix *M* in Eq. 1). This feature allows the user to investigate heterogeneity of samples and to identify potential outlier samples. Additionally, this information can be used to investigate correlation with clinical data including treatment history and survival outcomes.

The clustering of samples is performed using the k-means algorithm with Euclidian distance measure. For this purpose, the NbClust function from NbClust package^12^ in R is utilized which obtains the optimal number of clusters automatically based on 30 indices. CANCERSIGN produces two plots to visualize the clustering results. The first plot is the result of principal component analysis (PCA) of samples based on the features used for the clustering. The second plot shows the values of these features in the samples within each cluster by using box charts. As mentioned above, the features selected for clustering can be either the contribution of signatures to the mutational profiles of samples or the mutation counts within motifs selected by the user. The numeric values of the results produced by CANCERSIGN are stored in the output folder for further analyses (see CANCERSIGN manual).

We have applied our tool to the mutational data of 18 cancer types with sufficient number of samples that were available in the ICGC database, last updated June 2017 (Supplementary Table S1). General information and statistics about the data can be found in the Supplementary Table S1. Details about our analyses, including the tool parameters, are provided in the Supplementary Information File. We performed all the analyses with computational resources provided by the Telethon Kids Bioinformatics Server (30 CPU cores and 120 GB memory).

## Results and Discussion

**Figure 2:**
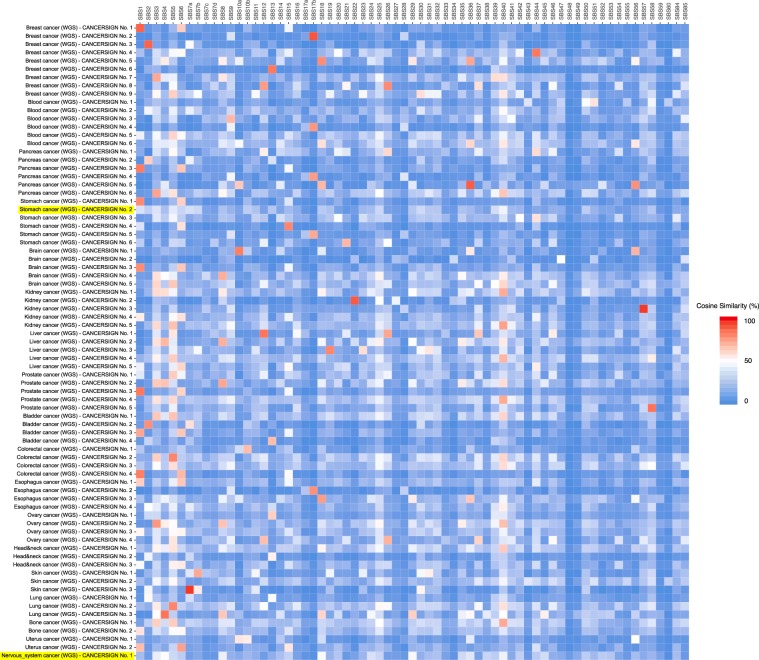
Correlation of 77 signatures identified by CANCERSIGN with 67 previously reported signatures by Alexandrov *et al*.^5^. Here, each cell indicates percentage of similarity between signatures identified by our tool and previously reported signatures. Those signatures with less than 50% similarity with previously reported signatures have been highlighted.

The results of separate analyses of whole genome mutation data from each of 18 tumor types reported in the ICGC database are presented in Supplementary Fig. S2A–R). The evaluation diagrams for each tumor type are presented in Supplementary Figs. S3–S20. These evaluation diagrams were used to determine the total number of mutational signatures presented in Supplementary Fig. S2. For each tumor type, the numerical values of the deciphered 3-mer signatures and the contribution of each signature to each tumor sample are provided in Supplementary Tables S2–S19. In total, CANCERSIGN identified 77 signatures across 18 tumor-types (Supplementary Fig. S2A–R). The majority of these signatures were highly similar (Cosine Similarity >50) to one or more of the recently reported 67 signatures by Alexandrov *et al*.^5^. But two of these signatures (one in stomach cancer and another one in nervous system cancer) had Cosine similarity of <50 with all the mentioned signatures, suggesting that they are possibly novel signatures that have not been identified by other studies so far (Fig. 2). For example, Supplementary Fig. S3 indicates nine mutational signatures deciphered from the mutational data of breast cancer samples using CANCERSIGN. For each of these nine signatures there is at least one previously reported signature which can be considered as a close match (Fig. 2). Supplementary Fig. S2D also shows the mutational signatures that CANCERSIGN has identified in stomach cancer samples. For this cancer type, one signature (Stomach CANCERSIGN Signature No. 2) does not resemble any of the previously reported signatures, i.e. it does not have >50% similarity to any of the previously reported COSMIC signatures (Fig. 2). Details of the correlations between all 77 signatures discovered by CANCERSIGN, and the signatures reported by Alexandrov *et al*.^5^ are given in the Supplementary Table S20. Hierarchical clustering of CANCERSIGN signatures based on their similarities to the Alexandrov signatures, revealed multiple CANCERSIGN signatures from different tumors, are highly similar to the Alexandrov signatures (Supplementary Fig. S26).

**Figure 3:**
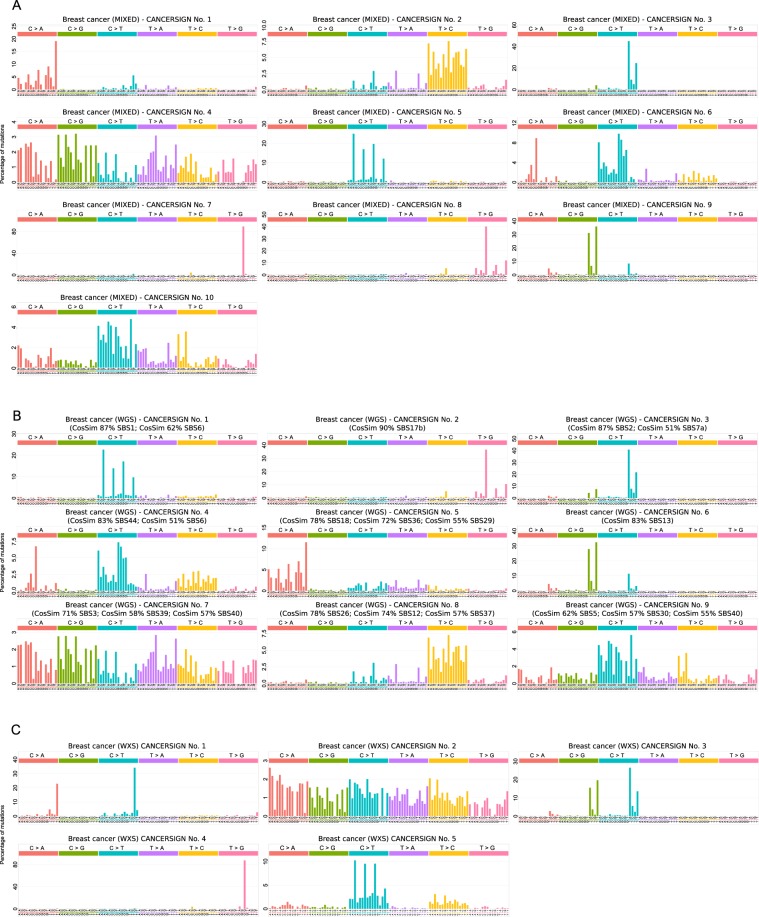
Mutational signatures deciphered from whole genome profiles, whole exome profiles and mixed (by pooling whole genome and whole exome) profiles of breast cancer samples. (**A)** For the mixed data. (**B)** For the whole genome data. (**C)** For the whole exome data.

**Figure 4:**
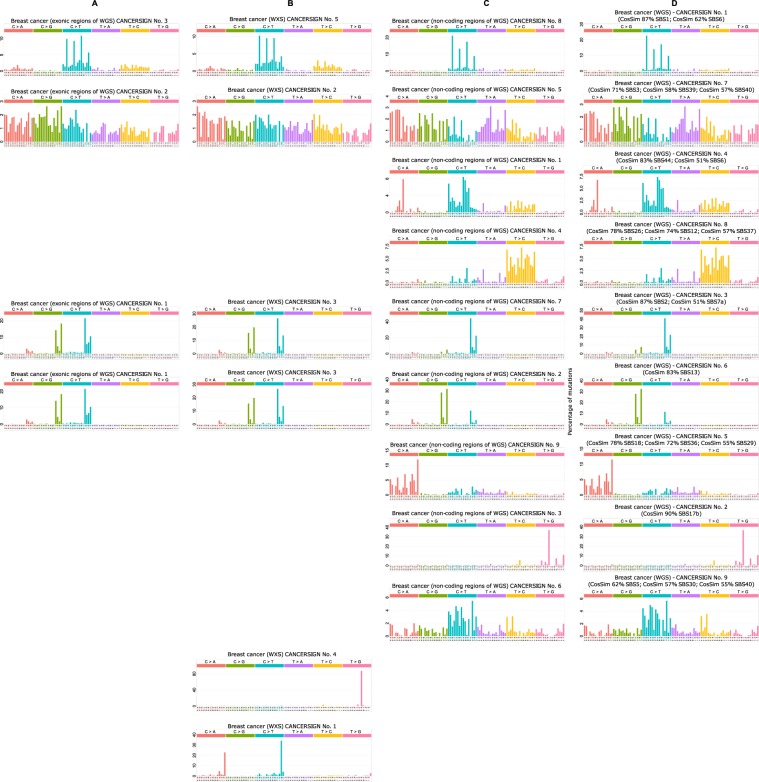
Mutational signatures deciphered from the whole genome profiles, whole exome profiles, exonic section of whole genome profiles and non-coding regions of whole genome profiles of breast cancer samples. (**A**) CANCERSIGN also analysed exonic sections of WGS breast cancer samples and identified three signatures. (**B)** In the analysis of whole exome sequencing breast cancer samples, CANCERSIGN detected five signatures. (**C)** In the analysis of non-coding regions of the WGS breast cancer samples, CANCERSIGN detected nine signatures, in which five of them seem to be non-coding specific signatures. (**D)** Nine signatures discovered from whole genome analysis of breast cancer sample by CNCERSIGN.

The 67 mutational signatures reported previously, is based on the analysis of all tumor types together. These signatures seem to be a consensus and represent trends across all tumor types. However, this global mutational signature analysis of multiple cancer types might produce results that are biased toward signatures of the tumors with more samples in the dataset. Furthermore, pooling somatic mutations obtained from whole exome sequencing with that of whole genome data can be another source of bias that is not considered in the previous studies. These issues have been discussed in a recent review by Nik-Zainal and Morganella^13^. Nevertheless, our tool can be used to extract mutational signatures for all tumors together, or for each tumor separately. Typically, researchers use mixed datasets (whole genome + whole exome) to extract mutational signatures. CANCERSIGN is able to provide an option for users to extract mutational signatures from the whole genome, whole exome or pooled data. Figure 3 shows the analysis of whole genome, whole exome and whole exome + whole genome (mixed) profiles of breast cancer samples (The evaluation diagrams for these analyses are presented in Supplementary Figs. S3, S21 and S22). For pooled data (WGS + WXS) we identified ten signatures (Fig. 3A), whereas for whole genome data we identified nine signatures (Fig. 3B**)**. The CANCERSIGN Signature No. 7 in Fig. 3A disappeared from the whole genome analysis and seems to be a WXS-specific signature. We then analysed breast cancer whole exome data and identified only five signatures (Fig. 3C). CANCERSIGN Signature No. 4 in Fig. 3C is identical to CANCERSIGN Signature No. 7 in Fig. 3A and CANCERSIGN Signature No. 1 in Fig. 3C has not appeared in either pooled or whole genome data. As a result, there are two signatures (No. 1 and No. 4 in Fig. 3C) in our analyses that seems to be associated with only whole exome data. To find out if this is a sequencing bias, we extracted exome parts of whole genome breast cancer samples; here, CANCERSIGN identified three signatures (Fig. 4A) that are identical with three of the five whole exome analysis signatures (No. 2, No. 3 and No.5 in Fig. 4B). CANCERSIGN Signatures No. 1 and No. 4 in Fig. 3C did not appear in this analysis, suggesting that these two signatures are specific for whole exome samples. Our detailed investigation of the breast cancer whole exome samples revealed that these samples have been sequenced ~2.5x more deeply than whole genome samples. This may indicate that the two WXS-specific signatures are rare signatures and can be seen in the deeply sequenced samples. We then extracted non-coding regions of the WGS breast cancer samples and used them as an input for CANCERSIGN. This analysis revealed nine signatures (Fig. 4C) that are identical to the results for the whole genome data (Fig. 4D); interestingly, five of the nine signatures did not appear in the analysis of the WXS samples or exonic regions of the WGS samples, suggesting that these five signatures are potentially specific to the mutations occurring in non-coding regions of the genome.

**Figure 5:**
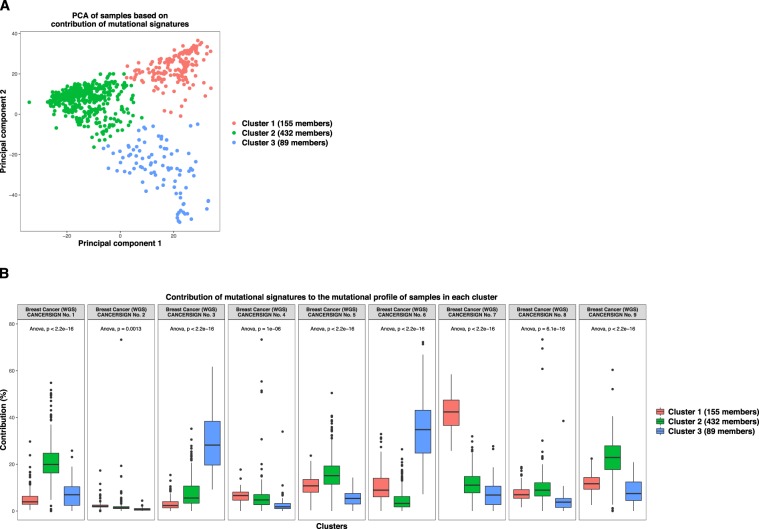
Clustering the samples of breast cancer using CANCERSIGN. (**A)** PCA of breast cancer tumor samples based on the contribution of 3-mer mutational signatures. The points are coloured based on clustering assignments. (**B)** Each box in the plot represents the contribution of one mutational signature to the mutational profiles of the samples within one cluster.

CANCERSIGN is also able to cluster tumor samples based on the contribution of mutational signatures to the mutational profile of samples or based on mutation counts in the profile of samples. This clustering analysis can be informative, because the results can be used along with clinical data such as subtype, response to therapies, and overall survival to identify potential biological associations. To demonstrate the clustering feature of CANCERSIGN, breast cancer samples were clustered based on the contribution of 3-mer mutational signatures deciphered in our whole genome analysis. The cluster assignments for the samples are provided in Supplementary Table S21 and the results are illustrated in Fig. 5. Figure 5A, which shows the PCA of samples based on the contribution of signatures, shows a clear separation among these clusters. Figure 5B provides more details about the contribution of these signatures to the profiles of samples within each cluster. According to this figure, CANCERSIGN Signature No. 7 in cluster 1 and CANCERSIGN Signatures No. 3 and No. 6 in cluster 3, have the highest contributions. This observation suggests that the mutational processes associated with these signatures may be potential biomarkers for these clusters.

**Figure 6:**
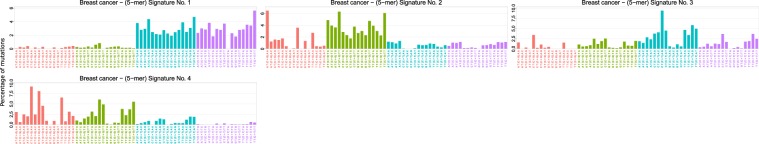
Deciphering whole genome 5-mer mutational signatures for breast cancer. The 5-mer mutational signatures extracted from whole-genome samples of breast cancer. Here, we have chosen four 3-mer motifs, namely T(C > G)A, T(C > G)T, T(C > T)A and T(C > T)T, to expand to 5-mer motifs and extracted the corresponding 5-mer signatures.

Another feature of CANCERSIGN is to decipher mutational signatures based on mutations within specific 5-mer motifs. To demonstrate this feature, we selected two 3-mer motifs (TCA and TCT) and two mutation types (C > T and C > G) as an input (i.e. T(C > G)A, T(C > G)T, T(C > T)A and T(C > T)T). These mutations are attributed to APOBEC enzymes and are the most abundant mutations in many cancer types^14,15^. CANSERSIGN first quantified the number of mutations within all possible 5-mer motifs that include the selected 3-mer motifs, then performed the mutational analysis for the resulting mutational profiles (for example, for C > G mutation within the 3-mer TCA motif, 16 possible 5-mers exists, i.e. NT(C > G)AN where N:A, C, G, or T). This process revealed four 5-mer mutational signatures as shown in Fig. 6. The evaluation diagram for this analysis is given in Supplementary Fig. S23. The numerical values of the 5-mer signatures and their contribution to the profiles of breast cancer samples are provided in Supplementary Table S22.

### Comparison with other tools

We have compared our tool with its well-known counterparts which have been developed for similar purposes: SomaticSignatures^7^, SigneR^8^ and deconstructSigs^9^. The deconstructSigs package is different from other tools as it is designed to determine the optimal linear combination of pre-defined mutational signatures that most accurately reconstructs the mutational profile of a single tumor sample^9^, whereas others are designed to extract *de novo* mutational signatures from a cohort. Table 1 compares the tools based on a set of features. Note that this comparison only considers relevant features of these tools rather than all capabilities. Please refer to the corresponding papers for more information^7–9^.

**Table 1:** A comparison between packages for analysis of mutational catalogues.

	Usage	Method of selecting optimum number of extracted signatures	Option of extracting 5-mer mutational signatures	Option of clustering the samples based on motifs or signatures	Implementation
CANCERSIGN	Deciphering *de novo* mutational signatures	Specified by user by inspecting the diagrams of reproducibility and reconstruction error	Yes	Yes	R
SomaticSignature	Deciphering *de novo* mutational signatures	Specified by user by inspecting the diagrams of residual sum of squares and explained variance	No	No	R
SigneR	Deciphering *de novo* mutational signatures	Automatically specified by empirical Bayesian treatment to the Poissonian NMF model	No	No	R
deconstructSigs	Obtain the optimal linear combination of pre-defined mutational signatures	—	No	No	R

The tools were applied to a simulated dataset of mutations. We constructed the simulated dataset as follows: 6 signatures were selected from the list of mutational signatures presented at the COSMIC database^6^ (signatures 1, 2, 3, 17, 21 and 29) as the underlying mutational processes, producing our simulated mutational catalogue. The mutational load (total mutations) for each sample was determined by taking a random number from a Rayleigh distribution with parameter $\sigma =8000$ (this distribution was used since we observed that the histogram of mutational loads for samples of a cancer is often unimodal and positively skewed, and the average of mutational loads across all samples and all cancer types is near 10’000 which is approximately equal to the mean of a Rayleigh distribution with scale parameter of 8000). In the next step, the vector of mutations for each sample was generated by a linear combination of the 6 signatures where the 6 coefficients were determined randomly (for each set of coefficients, we selected 6 random numbers between 0 to 1, then normalized them such that they sum to 1, and finally multiplied them with the amount of mutational load of the sample). The aforementioned algorithm was used to generate 400 samples to form the simulated mutational catalogue.

We tried to apply CANCERSIGN, SomaticSignatures and SigneR to the simulated dataset in an equal condition. However, SigneR, due to its Bayesian framework, is not scalable to large mutational catalogues containing several hundreds of samples. Consequently, it was not feasible to test SigneR on the simulation dataset and the comparison was made between CANCERSIGN and SomaticSignatures.

The parameters of the analysis were set as follows. The range of values of ***N*** (number of signatures to decipher) was set from 2 to 12. The maximum number of bootstraps for each ***N*** was set to 100 for CANCERSIGN, and the number of replicates (nReplicates) for SomaticSignatures was set to 20. With these settings, the tools consumed approximately the same amount of time (~45 minutes) to decipher mutational signatures from our simulated dataset (using a typical computer with four 1.7 GHz CPU cores and 8GB memory). According to Supplementary Fig. S24, both tools have correctly found ***N*** = **6** as the optimal number of underlying mutational signatures (the knee point in the diagram of summary statistics of SomaticSignatures^7^, and the point with a high reproducibility and the lowest reconstruction error in the evaluation diagram of CANCERSIGN). The obtained mutational signatures are shown in Supplementary Fig. S25. By a simple visual comparison, we can conclude that both tools have deciphered almost identical signatures which are also identical to the original selected signatures (signatures 1, 2, 3, 17, 21 and 29 from the COSMIC database). This test shows that both tools can produce valid results when analysing catalogues of somatic signatures.

We also compared the tools in terms of time efficiency. Note that the number of replicates for SomaticSignatures is equivalent to the number of bootstraps for CANCERSIGN. It is observed that in the same amount of time (~45 minutes), the number of bootstraps performed by CANCERSIGN was more than the number of replicates in the process of SomaticSignatures (100 vs. 20). Thus, we can conclude that CANCERSIGN performs the analyses faster than SomaticSignatures.

## Conclusion

Systematic analyses of mutations in tumor biopsies from a large number of cancer types have identified at least 67 mutational signatures, each pointing to distinct molecular mechanisms acting on cellular DNA. The computational method used to extract mutational signatures is becoming an integral part of cancer research. Therefore, in recent years, a number of studies have focused specifically on the development of bioinformatics tools for analysis of mutational signatures. In the present study, we reported the development of a stand-alone tool called CANCERSIGN, which does not require any programming skills to be used. This tool has several unique features. Firstly, it has been optimized to run parallel computational analysis in order to speed up *de novo* mutational signature extraction. Secondly, it enables the extraction of 5-mer mutational signature profiles in addition to the commonly used 3-mer signature patterns. Thirdly, using the clustering features of this tool, differences between patients and/or tumor types can be investigated based on the contribution of mutational signatures to the mutational profiles of samples or based on their mutation counts within specific motifs. In addition, using this tool, we identified a number of novel signatures. Overall, CANCERSIGN is a multi-functional and user-friendly computational tool for accurate and quick analysis of mutation signatures and clustering of tumor samples. This tool is a stand-alone package, is freely available, and does not require any specific computational skills to run. We’ve previously shown that Markov model of probabilities can be used to quantify the “representation” of motifs (e.g. D-ratio) and so to distinguish under-represented and over-represented motifs in HIV-1 and human genomes^16,17^. As an interesting future study, we plan to use D-ratio to re-implement NMF algorithm and use the new algorithm to accurately identify cancer mutational signatures without using alignment.

## Data availibility

All supplementary files are available on the journal website.

## Supplementary information

Supplementary files.

Supplementary tables.
