# Supplementary material for: CANCERSIGN: a user-friendly and robust tool for identification and classification of mutational signatures and patterns in cancer genomes
Source: Sci Rep. 2020 Jan 28;10:1286. doi: 10.1038/s41598-020-58107-2 (PMC6987109; doi:10.1038/s41598-020-58107-2)
Supplement: Supplementary file 1 — Supplementary files. [file 41598_2020_58107_MOESM1_ESM.pdf]

CANCERSIGN: a user-friendly and robust tool for identification and classification of mutational signatures and patterns in cancer genomes

Masroor Bayati[1], Hamid R. Rabiee[#1], Mehrdad Mehrbod[1], Fatemeh Vafaee[2], Diako Ebrahimi[3], Alistair R.R. Forrest[4], Hamid Alinejad-Rokny[*4,5]

[1]Bioinformatics and Computational Biology Lab, Department of Computer Engineering, Sharif University of Technology, Tehran, 11365, IR

[2]School of Biotechnology and Biomolecular Sciences, University of New South Wales, UNSW Sydney, 2033, NSW, AU

[3]Quantitative Biology Lab, Texas Biomedical Research Institute, Texas, TX, US

[4]Systems Biology and Health Data Analytics Lab, The Graduate School of Biomedical Engineering, UNSW Australia, Sydney, 2052, AU

[5]Harry Perkins Institute of Medical Research, QEII Medical Centre and Centre for Medical Research, The University of Western Australia, Nedlands 6009, AU

* To whom correspondence should be addressed. Tel: +61 2 9385 3911; E-mail: h.alinejad@ieee.org.

HRR is the second corresponding author of this paper.

ALGORITHM PARAMETERS

For deciphering 3-mer and 5-mer mutational signatures from the samples of each cancer type (Figures 2 and 3), the algorithm parameters are set as follows: number of iterations for NMF algorithm in each epoch is set to 10’000 (parameter: nmf_iters). These iterations are repeated until convergence which is determined by checking that after the last NMF epoch, the maximum change in the elements of signatures matrix (*P*) is less than 10^{-5} (parameter: nmf_conv). In this analysis, the maximum number of NMF iterations is 500’000 (parameter: nmf_total_max). For each value of ***N***, the NMF procedure is repeatedly performed for bootstrapped catalogue matrix in several epochs. In each epoch, at least 30 bootstraps (parameter: $boot\_iters$) are performed (if the number of available CPU cores is more than 30, then this number is considered for this parameter) and the epochs are repeated until the deciphered signatures become stable. This stopping point is recognized when the cosine distance between signatures deciphered up to current epoch and the signatures deciphered after doubling the bootstrapping iterations is less than 10^{-2} (parameter: $boot\_conv$). Finally, the maximum number of bootstrapping repeats in this analysis is set to 600 (parameter: $boot\_total\_max$).

Note that the aforementioned parameters are set to extreme values in order to obtain precise results for publication purposes. In many situations, we have observed that the overall shape of the solutions (number of signatures and their distribution over motifs) are revealed and stabilized by more relaxed parameters (such as 50’000 and 100 for nmf_total_max and $boot\_total\_max$ parameters, respectively).

SUPPLEMENTARY FIGURES LEGENDS

Figure S1 | CANCERSIGN in action. This tool can be used without need for programming skills in R. The user only needs to provide the input file (mutational catalogue of tumor samples with the valid format described in the tool manual) and set the appropriate configurations for the tool by manually editing the “configuration file”. In short, the configurations include path to the input directory, path to the output directory, type of analysis and analysis accuracy parameters.

Figure S2 | Deciphering whole genome 3-mer mutational signatures for various cancer types. The 3-mer mutational signatures extracted from whole-genome samples of 18 cancer types. Each cancer type is analysed separately. **(A)** Breast cancer 3-mer signatures. **(B)** Brain cancer 3-mer signatures. **(C)** Esophagus cancer 3-mer signatures. **(R)** Nervous system cancer 3-mer signatures.

Figure S3 | Evaluation plot for deciphering 3-mer mutational signatures. Whole genome samples in breast cancer.

Figure S4 | Evaluation plot for deciphering 3-mer mutational signatures. Whole genome samples in blood cancer.

Figure S5 | Evaluation plot for deciphering 3-mer mutational signatures. Whole genome samples in pancreas cancer.

Figure S6 | Evaluation plot for deciphering 3-mer mutational signatures. Whole genome samples in stomach cancer.

Figure S7 | Evaluation plot for deciphering 3-mer mutational signatures. Whole genome samples in brain cancer.

Figure S8 | Evaluation plot for deciphering 3-mer mutational signatures. Whole genome samples in kidney cancer.

Figure S9 | Evaluation plot for deciphering 3-mer mutational signatures. Whole genome samples in liver cancer.

Figure S10 | Evaluation plot for deciphering 3-mer mutational signatures. Whole genome samples in prostate cancer.

Figure S11 | Evaluation plot for deciphering 3-mer mutational signatures. Whole genome samples in bladder cancer.

Figure S12 | Evaluation plot for deciphering 3-mer mutational signatures. Whole genome samples in colorectal cancer.

Figure S13 | Evaluation plot for deciphering 3-mer mutational signatures. Whole genome samples in Oesophagus cancer.

Figure S14 | Evaluation plot for deciphering 3-mer mutational signatures. Whole genome samples in ovary cancer.

Figure S15 | Evaluation plot for deciphering 3-mer mutational signatures. Whole genome samples in head & neck cancer.

Figure S16 | Evaluation plot for deciphering 3-mer mutational signatures. Whole genome samples in skin cancer.

Figure S17 | Evaluation plot for deciphering 3-mer mutational signatures. Whole genome samples in lung cancer.

Figure S18 | Evaluation plot for deciphering 3-mer mutational signatures. Whole genome samples in bone cancer.

Figure S19 | Evaluation plot for deciphering 3-mer mutational signatures. Whole genome samples in uterus cancer.

Figure S20 | Evaluation plot for deciphering 3-mer mutational signatures. Whole genome samples in nervous system cancer.

Figure S21 | Evaluation plot for deciphering 3-mer mutational signatures. Whole exome samples in breast cancer.

Figure S22 | Evaluation plot for deciphering 3-mer mutational signatures. Mixed data (by pooling whole genome and whole exome) samples in breast cancer.

Figure S23 | Evaluation plot for deciphering 5-mer mutational signatures. Whole genome samples in breast cancer.

Figure S24 | **Evaluation diagrams produced by SomaticSignatures and CANCERSIGN packages when applied to the sample dataset (for tool comparison).**

(A) For SomaticSignatures package. **(B)** For CANCERSIGN package.

Figure S25 | Mutational signatures deciphered by SomaticSignatures and CANCERSIGN packages when applied to the sample dataset (for tool comparison). (A) For the SomaticSignatures package. **(B)** For the CANCERSIGN package.

SUPPLEMENTARY TABLES LEGENDS

Table S1 | General statistics of the dataset of somatic mutations.

Table S2 | Numerical values of deciphered 3-mer mutational signatures. Whole genome samples in breast cancer.

Table S3 | Numerical values of deciphered 3-mer mutational signatures. Whole genome samples in blood cancer.

Table S4 | Numerical values of deciphered 3-mer mutational signatures. Whole genome samples in pancreas cancer.

Table S5 | Numerical values of deciphered 3-mer mutational signatures. Whole genome samples in stomach cancer.

Table S6 | Numerical values of deciphered 3-mer mutational signatures. Whole genome samples in brain cancer.

Table S7 | Numerical values of deciphered 3-mer mutational signatures. Whole genome samples in kidney cancer.

Table S8 | Numerical values of deciphered 3-mer mutational signatures. Whole genome samples in liver cancer.

Table S9 | Numerical values of deciphered 3-mer mutational signatures. Whole genome samples in prostate cancer.

Table S10 | Numerical values of deciphered 3-mer mutational signatures. Whole genome samples in bladder cancer.

Table S11 | Numerical values of deciphered 3-mer mutational signatures. Whole genome samples in colorectal cancer.

Table S12 | Numerical values of deciphered 3-mer mutational signatures. Whole genome samples in esophagus cancer.

Table S13 | Numerical values of deciphered 3-mer mutational signatures. Whole genome samples in ovary cancer.

Table S14 | Numerical values of deciphered 3-mer mutational signatures. Whole genome samples in head & neck cancer.

Table S15 | Numerical values of deciphered 3-mer mutational signatures. Whole genome samples in skin cancer.

Table S16 | Numerical values of deciphered 3-mer mutational signatures. Whole genome samples in lung cancer.

Table S17 | Numerical values of deciphered 3-mer mutational signatures. Whole genome samples in bone cancer.

Table S18 | Numerical values of deciphered 3-mer mutational signatures. Whole genome samples in uterus cancer.

Table S19 | Numerical values of deciphered 3-mer mutational signatures. Whole genome samples in nervous system cancer.

Table S20 | Numerical values of correlation of 77 signatures identified by CANCERSIGN with 30 previously reported signatures by Nik-Zainal.

Table S21 | Result of clustering breast cancer samples. Cluster assignments for samples is provided in this table. This clustering is carried out based on the contribution of 3-mer mutational signatures that CANCERSIGN has deciphered from whole-genome breast cancer samples.

Table S22 | Numerical values of deciphered 5-mer mutational signatures. Whole genome samples in breast cancer.

Figure S1

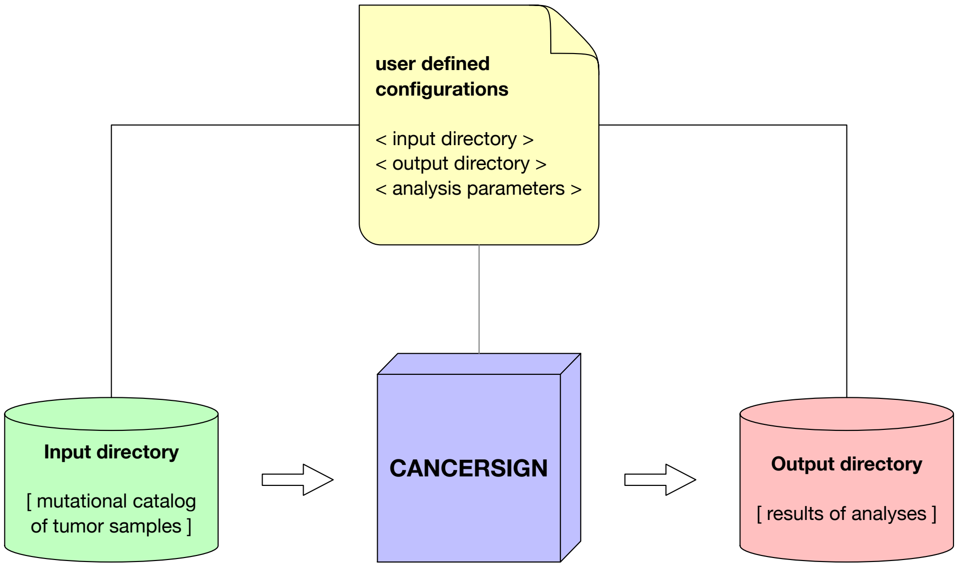
user defined
configurations
< input directory >
< output directory >
< analysis parameters >
Input directory
[mutational catalog
of tumor samples]
CANCERSIGN
Output directory
[results of analyses]

Figure S2-1

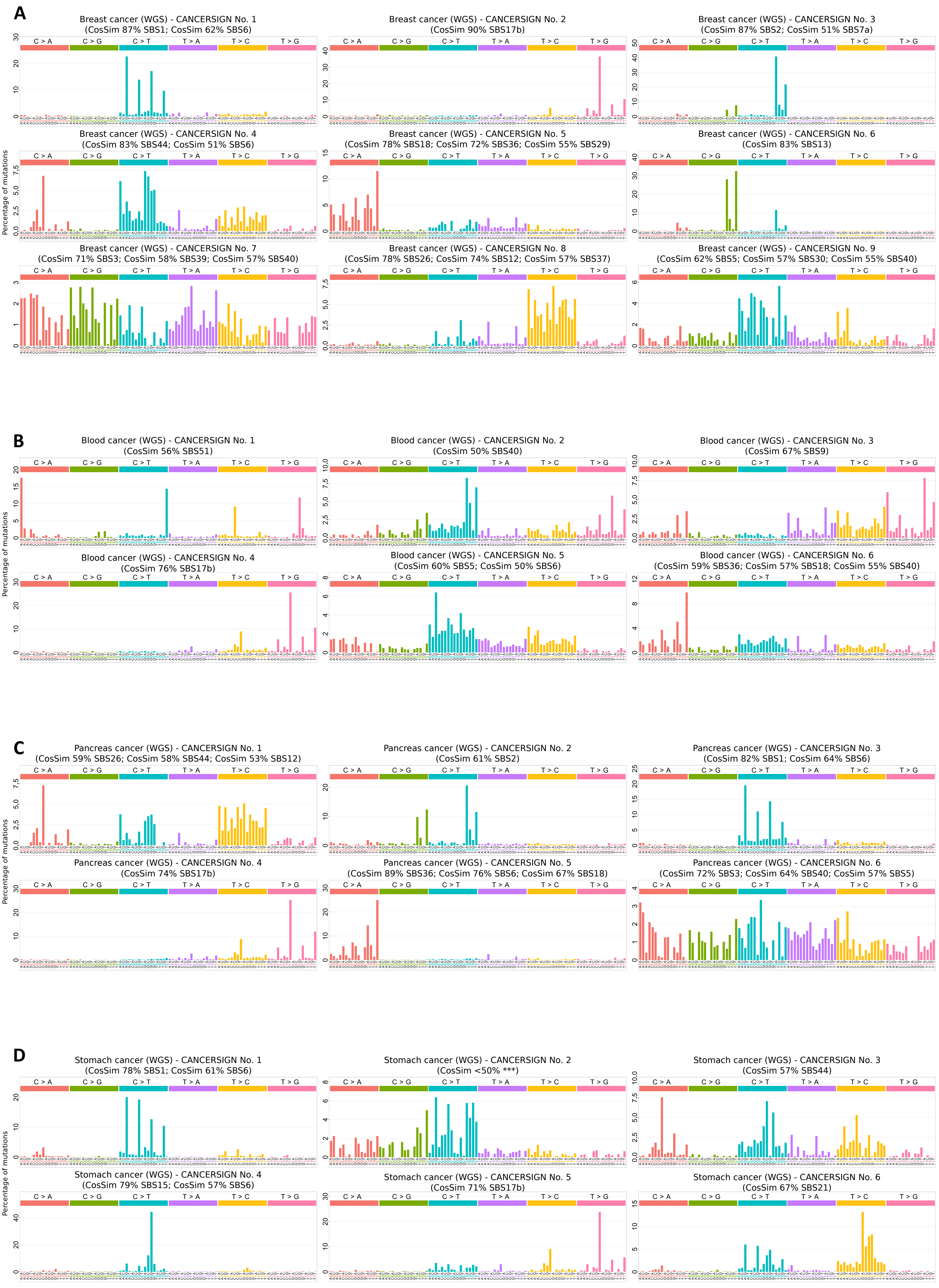
A
Breast cancer (WGS) - CANCERSIGN No. 1
(CosSim 87% SBS1; CosSim 62% SBS6)
Breast cancer (WGS) - CANCERSIGN No. 2
(CosSim 90% SBS17b)
Breast cancer (WGS) - CANCERSIGN No. 3
(CosSim 87% SBS2; CosSim 51% SBS7a)
Breast cancer (WGS) - CANCERSIGN No. 4
(CosSim 83% SBS44; CosSim 51% SBS6)
Breast cancer (WGS) - CANCERSIGN No. 5
(CosSim 78% SBS18; CosSim 72% SBS36; CosSim 55% SBS29)
Breast cancer (WGS) - CANCERSIGN No. 6
(CosSim 83% SBS13)
Breast cancer (WGS) - CANCERSIGN No. 7
(CosSim 71% SBS3; CosSim 58% SBS39; CosSim 57% SBS40)
Breast cancer (WGS) - CANCERSIGN No. 8
(CosSim 78% SBS26; CosSim 74% SBS12; CosSim 57% SBS37)
Breast cancer (WGS) - CANCERSIGN No. 9
(CosSim 62% SBS5; CosSim 57% SBS30; CosSim 55% SBS40)
B
Blood cancer (WGS) - CANCERSIGN No. 1
(CosSim 56% SBS51)
Blood cancer (WGS) - CANCERSIGN No. 2
(CosSim 50% SBS40)
Blood cancer (WGS) - CANCERSIGN No. 3
(CosSim 67% SBS9)
Blood cancer (WGS) - CANCERSIGN No. 4
(CosSim 76% SBS17b)
Blood cancer (WGS) - CANCERSIGN No. 5
(CosSim 60% SBS5; CosSim 50% SBS6)
Blood cancer (WGS) - CANCERSIGN No. 6
(CosSim 59% SBS36; CosSim 57% SBS18; CosSim 55% SBS40)
C
Pancreas cancer (WGS) - CANCERSIGN No. 1
(CosSim 59% SBS26; CosSim 58% SBS44; CosSim 53% SBS12)
Pancreas cancer (WGS) - CANCERSIGN No. 2
(CosSim 61% SBS2)
Pancreas cancer (WGS) - CANCERSIGN No. 3
(CosSim 82% SBS1; CosSim 64% SBS6)
Pancreas cancer (WGS) - CANCERSIGN No. 4
(CosSim 74% SBS17b)
Pancreas cancer (WGS) - CANCERSIGN No. 5
(CosSim 89% SBS36; CosSim 76% SBS6; CosSim 67% SBS18)
Pancreas cancer (WGS) - CANCERSIGN No. 6
(CosSim 72% SBS3; CosSim 64% SBS40; CosSim 57% SBS5)
D
Stomach cancer (WGS) - CANCERSIGN No. 1
(CosSim 78% SBS1; CosSim 61% SBS6)
Stomach cancer (WGS) - CANCERSIGN No. 2
(CosSim <50% ***)
Stomach cancer (WGS) - CANCERSIGN No. 3
(CosSim 57% SBS44)
Stomach cancer (WGS) - CANCERSIGN No. 4
(CosSim 79% SBS15; CosSim 57% SBS6)
Stomach cancer (WGS) - CANCERSIGN No. 5
(CosSim 71% SBS17b)
Stomach cancer (WGS) - CANCERSIGN No. 6
(CosSim 67% SBS21)
Percentage of mutations
C > A
C > G
C > T
T > A
T > C
T > G

Figure S2-2

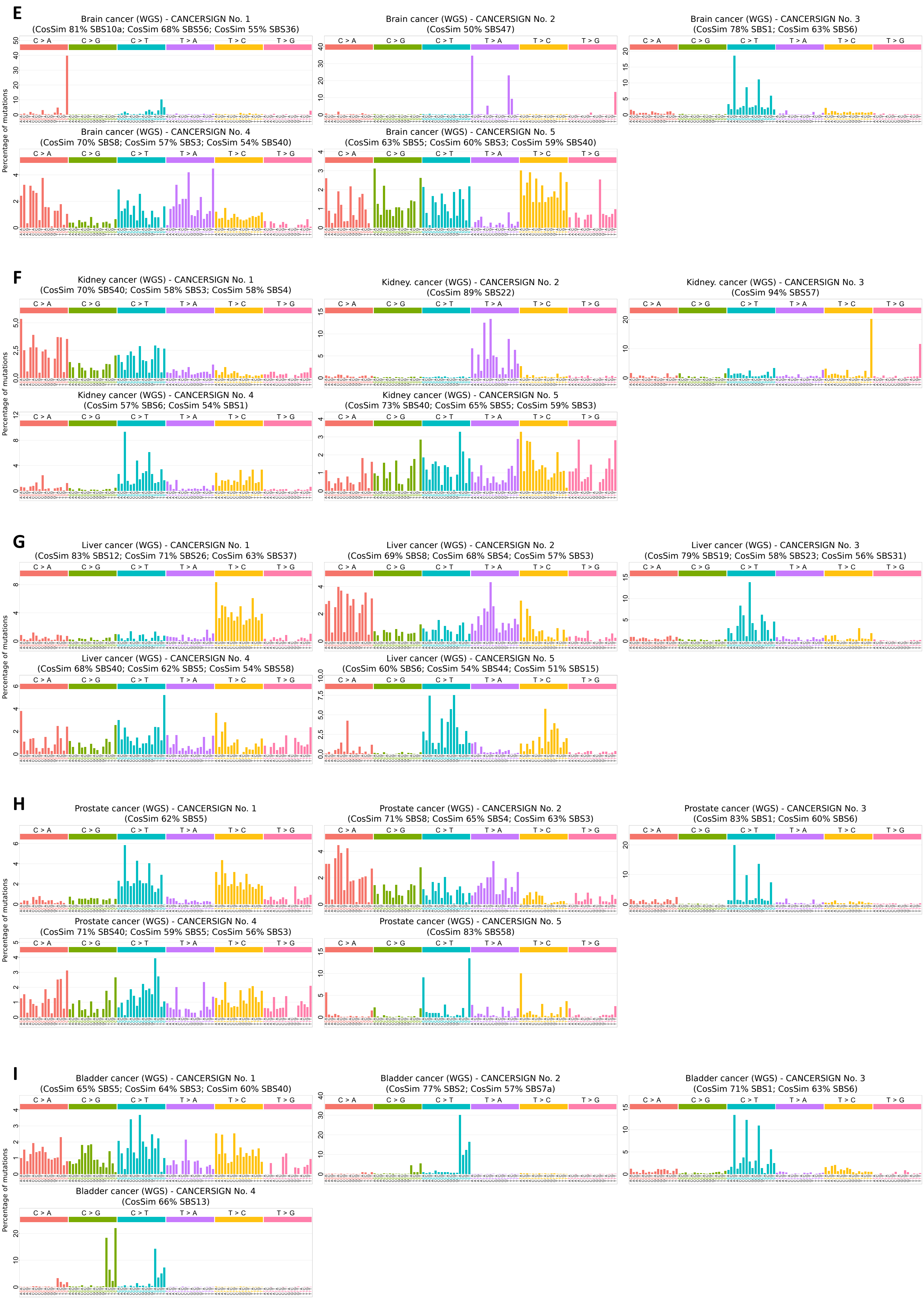
E
Brain cancer (WGS) - CANCERSIGN No. 1
(CosSim 81% SBS10a; CosSim 68% SBS56; CosSim 55% SBS36)
Brain cancer (WGS) - CANCERSIGN No. 2
(CosSim 50% SBS47)
Brain cancer (WGS) - CANCERSIGN No. 3
(CosSim 78% SBS1; CosSim 63% SBS6)
Brain cancer (WGS) - CANCERSIGN No. 4
(CosSim 70% SBS8; CosSim 57% SBS3; CosSim 54% SBS40)
Brain cancer (WGS) - CANCERSIGN No. 5
(CosSim 63% SBS5; CosSim 60% SBS3; CosSim 59% SBS40)
F
Kidney cancer (WGS) - CANCERSIGN No. 1
(CosSim 70% SBS40; CosSim 58% SBS3; CosSim 58% SBS4)
Kidney. cancer (WGS) - CANCERSIGN No. 2
(CosSim 89% SBS22)
Kidney. cancer (WGS) - CANCERSIGN No. 3
(CosSim 94% SBS57)
Kidney cancer (WGS) - CANCERSIGN No. 4
(CosSim 57% SBS6; CosSim 54% SBS1)
Kidney cancer (WGS) - CANCERSIGN No. 5
(CosSim 73% SBS40; CosSim 65% SBS5; CosSim 59% SBS3)
G
Liver cancer (WGS) - CANCERSIGN No. 1
(CosSim 83% SBS12; CosSim 71% SBS26; CosSim 63% SBS37)
Liver cancer (WGS) - CANCERSIGN No. 2
(CosSim 69% SBS8; CosSim 68% SBS4; CosSim 57% SBS3)
Liver cancer (WGS) - CANCERSIGN No. 3
(CosSim 79% SBS19; CosSim 58% SBS23; CosSim 56% SBS31)
Liver cancer (WGS) - CANCERSIGN No. 4
(CosSim 68% SBS40; CosSim 62% SBS5; CosSim 54% SBS58)
Liver cancer (WGS) - CANCERSIGN No. 5
(CosSim 60% SBS6; CosSim 54% SBS44; CosSim 51% SBS15)
H
Prostate cancer (WGS) - CANCERSIGN No. 1
(CosSim 62% SBS5)
Prostate cancer (WGS) - CANCERSIGN No. 2
(CosSim 71% SBS8; CosSim 65% SBS4; CosSim 63% SBS3)
Prostate cancer (WGS) - CANCERSIGN No. 3
(CosSim 83% SBS1; CosSim 60% SBS6)
Prostate cancer (WGS) - CANCERSIGN No. 4
(CosSim 71% SBS40; CosSim 59% SBS5; CosSim 56% SBS3)
Prostate cancer (WGS) - CANCERSIGN No. 5
(CosSim 83% SBS58)
I
Bladder cancer (WGS) - CANCERSIGN No. 1
(CosSim 65% SBS5; CosSim 64% SBS3; CosSim 60% SBS40)
Bladder cancer (WGS) - CANCERSIGN No. 2
(CosSim 77% SBS2; CosSim 57% SBS7a)
Bladder cancer (WGS) - CANCERSIGN No. 3
(CosSim 71% SBS1; CosSim 63% SBS6)
Bladder cancer (WGS) - CANCERSIGN No. 4
(CosSim 66% SBS13)
Percentage of mutations
C > A
C > G
C > T
T > A
T > C
T > G

Figure S2-3

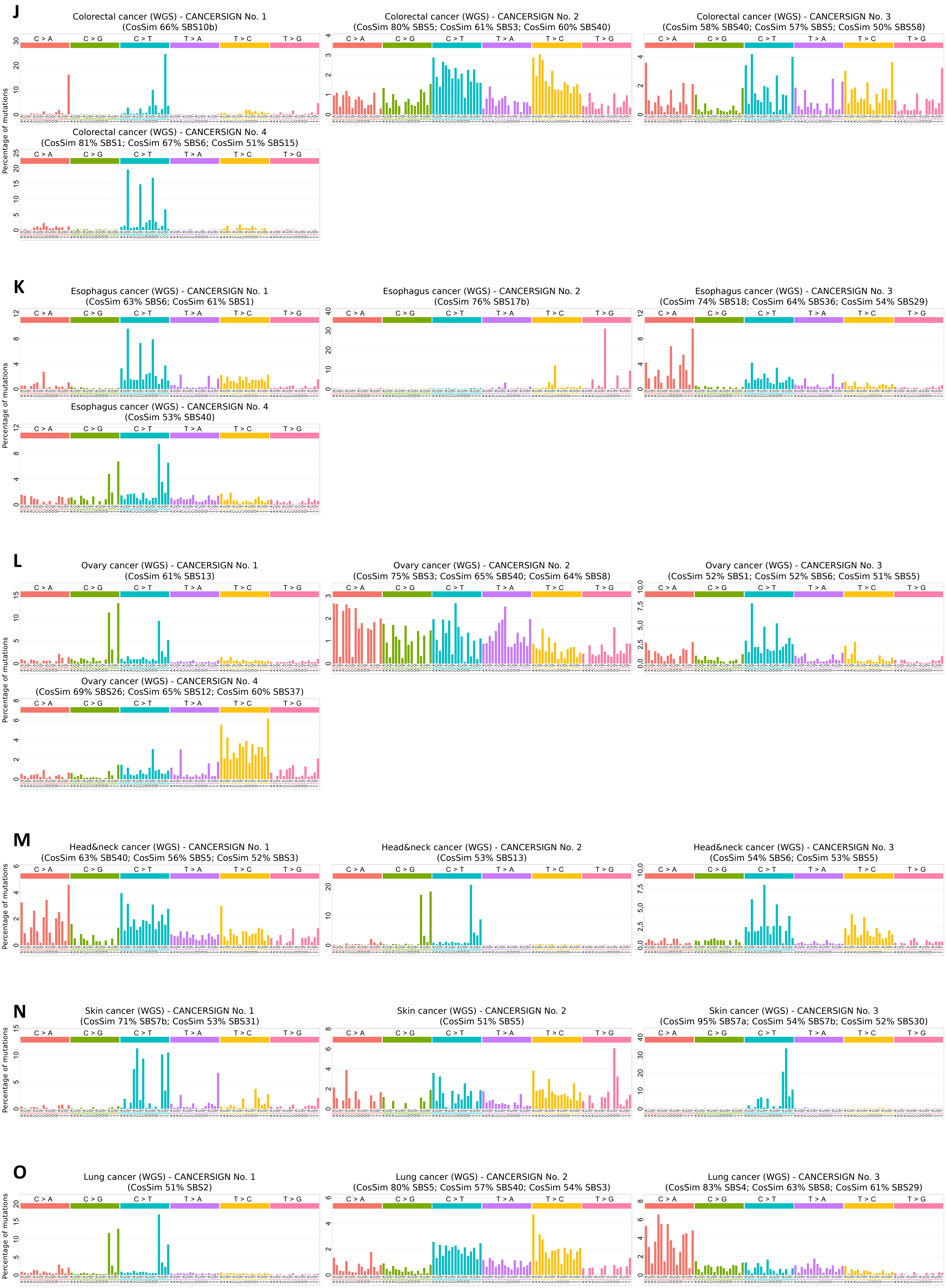

J
Colorectal cancer (WGS) - CANCERSIGN No. 1
(CosSim 66% SBS10b)
Colorectal cancer (WGS) - CANCERSIGN No. 2
(CosSim 80% SBS5; CosSim 61% SBS3; CosSim 60% SBS40)
Colorectal cancer (WGS) - CANCERSIGN No. 3
(CosSim 58% SBS40; CosSim 57% SBS5; CosSim 50% SBS58)
Colorectal cancer (WGS) - CANCERSIGN No. 4
(CosSim 81% SBS1; CosSim 67% SBS6; CosSim 51% SBS15)
K
Esophagus cancer (WGS) - CANCERSIGN No. 1
(CosSim 63% SBS6; CosSim 61% SBS1)
Esophagus cancer (WGS) - CANCERSIGN No. 2
(CosSim 76% SBS17b)
Esophagus cancer (WGS) - CANCERSIGN No. 3
(CosSim 74% SBS18; CosSim 64% SBS36; CosSim 54% SBS29)
Esophagus cancer (WGS) - CANCERSIGN No. 4
(CosSim 53% SBS40)
L
Ovary cancer (WGS) - CANCERSIGN No. 1
(CosSim 61% SBS13)
Ovary cancer (WGS) - CANCERSIGN No. 2
(CosSim 75% SBS3; CosSim 65% SBS40; CosSim 64% SBS8)
Ovary cancer (WGS) - CANCERSIGN No. 3
(CosSim 52% SBS1; CosSim 52% SBS6; CosSim 51% SBS5)
Ovary cancer (WGS) - CANCERSIGN No. 4
(CosSim 69% SBS26; CosSim 65% SBS12; CosSim 60% SBS37)
M
Head&neck cancer (WGS) - CANCERSIGN No. 1
(CosSim 63% SBS40; CosSim 56% SBS5; CosSim 52% SBS3)
Head&neck cancer (WGS) - CANCERSIGN No. 2
(CosSim 53% SBS13)
Head&neck cancer (WGS) - CANCERSIGN No. 3
(CosSim 54% SBS6; CosSim 53% SBS5)
N
Skin cancer (WGS) - CANCERSIGN No. 1
(CosSim 71% SBS7b; CosSim 53% SBS31)
Skin cancer (WGS) - CANCERSIGN No. 2
(CosSim 51% SBS5)
Skin cancer (WGS) - CANCERSIGN No. 3
(CosSim 95% SBS7a; CosSim 54% SBS7b; CosSim 52% SBS30)
O
Lung cancer (WGS) - CANCERSIGN No. 1
(CosSim 51% SBS2)
Lung cancer (WGS) - CANCERSIGN No. 2
(CosSim 80% SBS5; CosSim 57% SBS40; CosSim 54% SBS3)
Lung cancer (WGS) - CANCERSIGN No. 3
(CosSim 83% SBS4; CosSim 63% SBS8; CosSim 61% SBS29)
C > A
C > G
C > T
T > A
T > C
T > G
Percentage of mutations

Figure S2-4

P

Bone cancer (WGS) - CANCERSIGN No. 1
(CosSim 71% SBS40; CosSim 62% SBS3; CosSim 62% SBS5)

Bone cancer (WGS) - CANCERSIGN No. 2
(CosSim 56% SBS1; CosSim 51% SBS6)

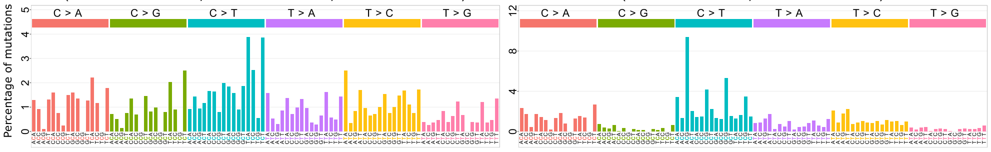

Q

Uterus cancer (WGS) - CANCERSIGN No. 1
(CosSim 55% SBS10a; CosSim 54% SBS10b; CosSim 50% SBS56)

Uterus cancer (WGS) - CANCERSIGN No. 2
(CosSim 73% SBS6; CosSim 66% SBS1; CosSim 56% SBS15)

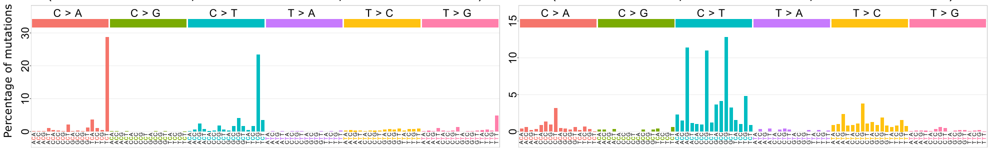

R

Nervous_system cancer (WGS) - CANCERSIGN No. 1
(CosSim <50% ***)

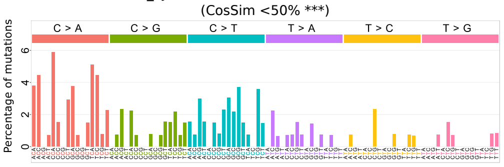

Figure S3

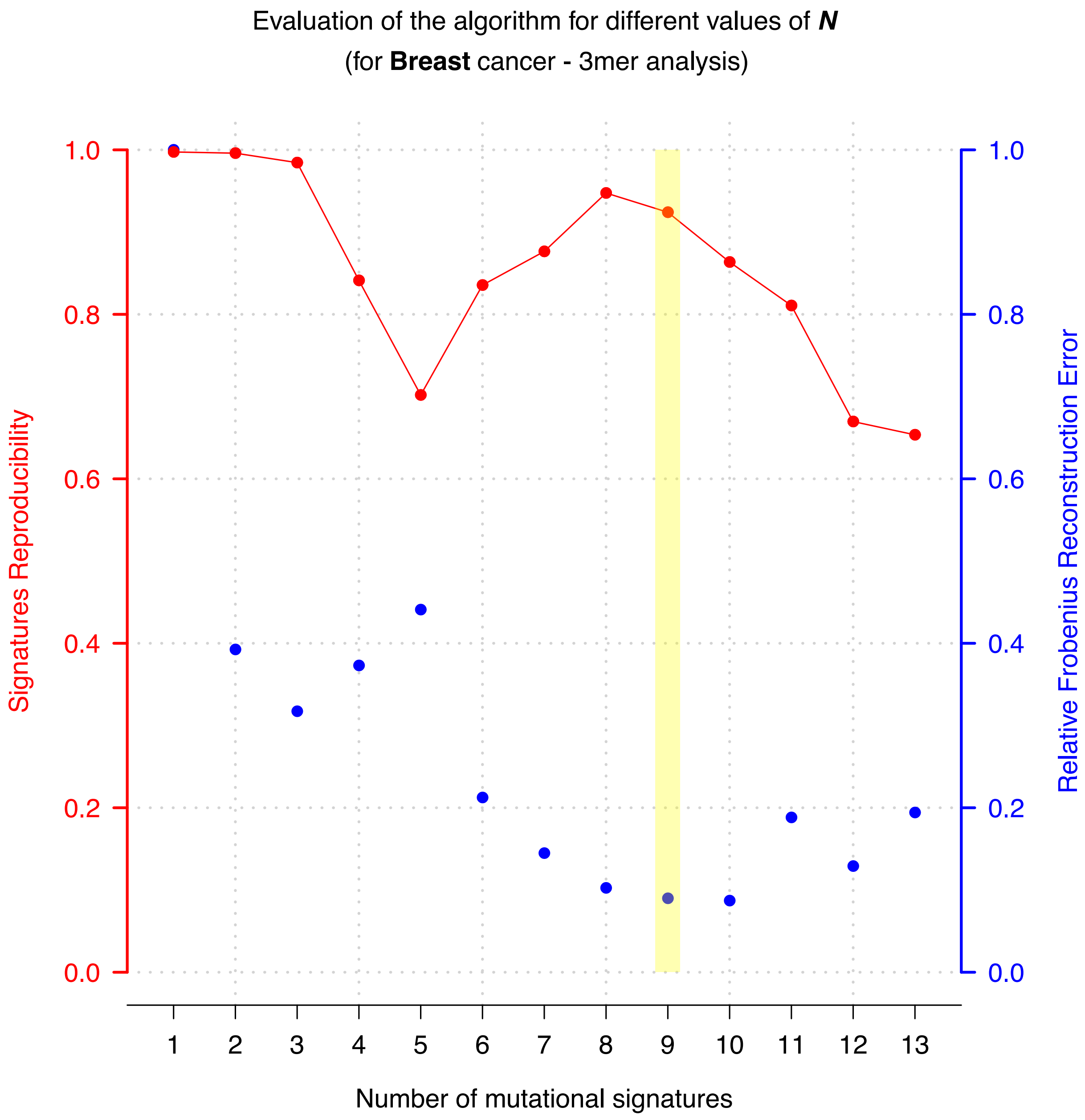

Evaluation of the algorithm for different values of ***N***
(for **Breast** cancer - 3mer analysis)
Signatures Reproducibility
Relative Frobenius Reconstruction Error
Number of mutational signatures
1.0
0.8
0.6
0.4
0.2
0.0
1
2
3
4
5
6
7
8
9
10
11
12
13

Figure S4

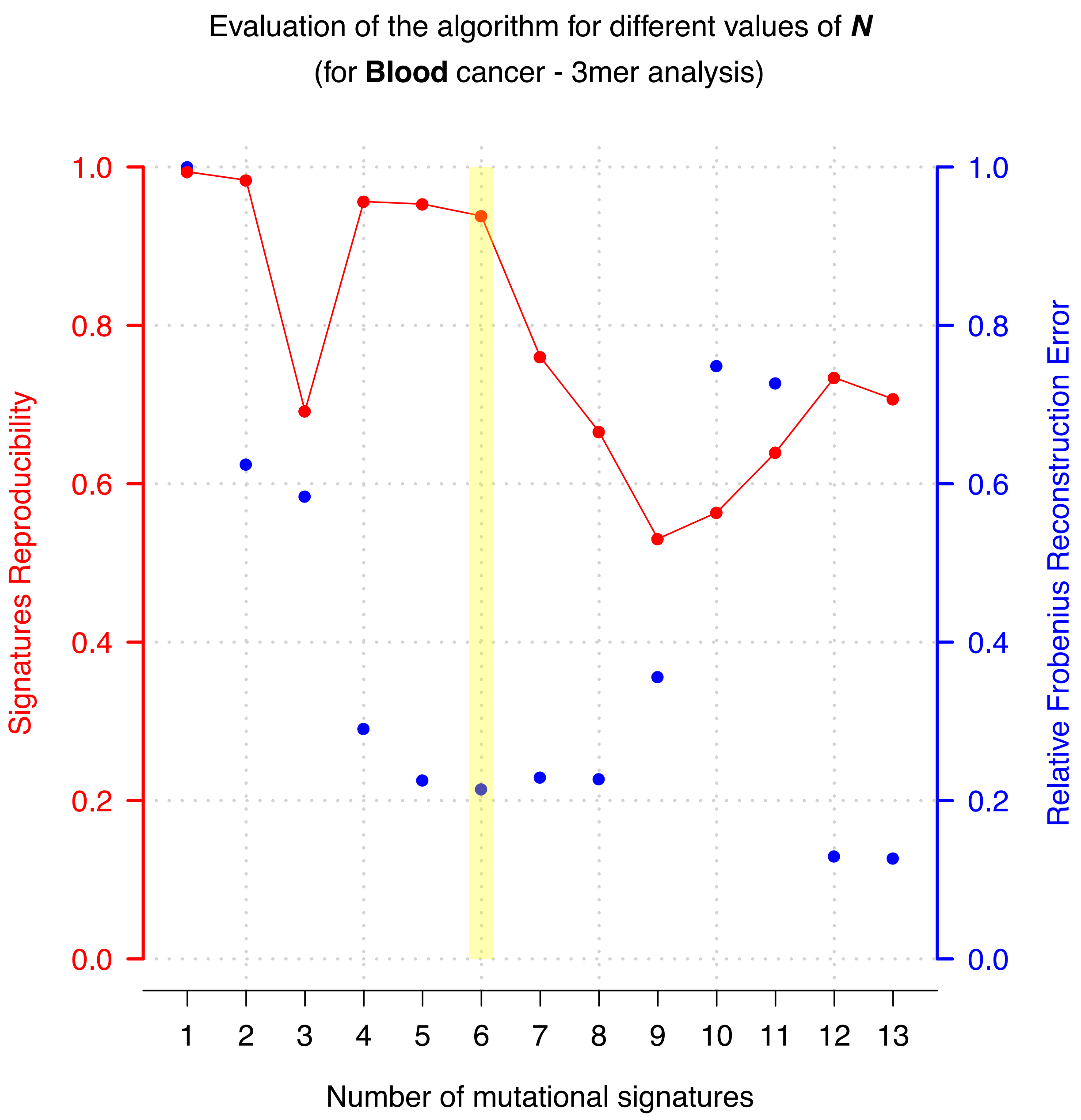
Evaluation of the algorithm for different values of ***N***
(for **Blood** cancer - 3mer analysis)
Signatures Reproducibility
Relative Frobenius Reconstruction Error
1.0
0.8
0.6
0.4
0.2
0.0
1
2
3
4
5
6
7
8
9
10
11
12
13
Number of mutational signatures

Figure S5

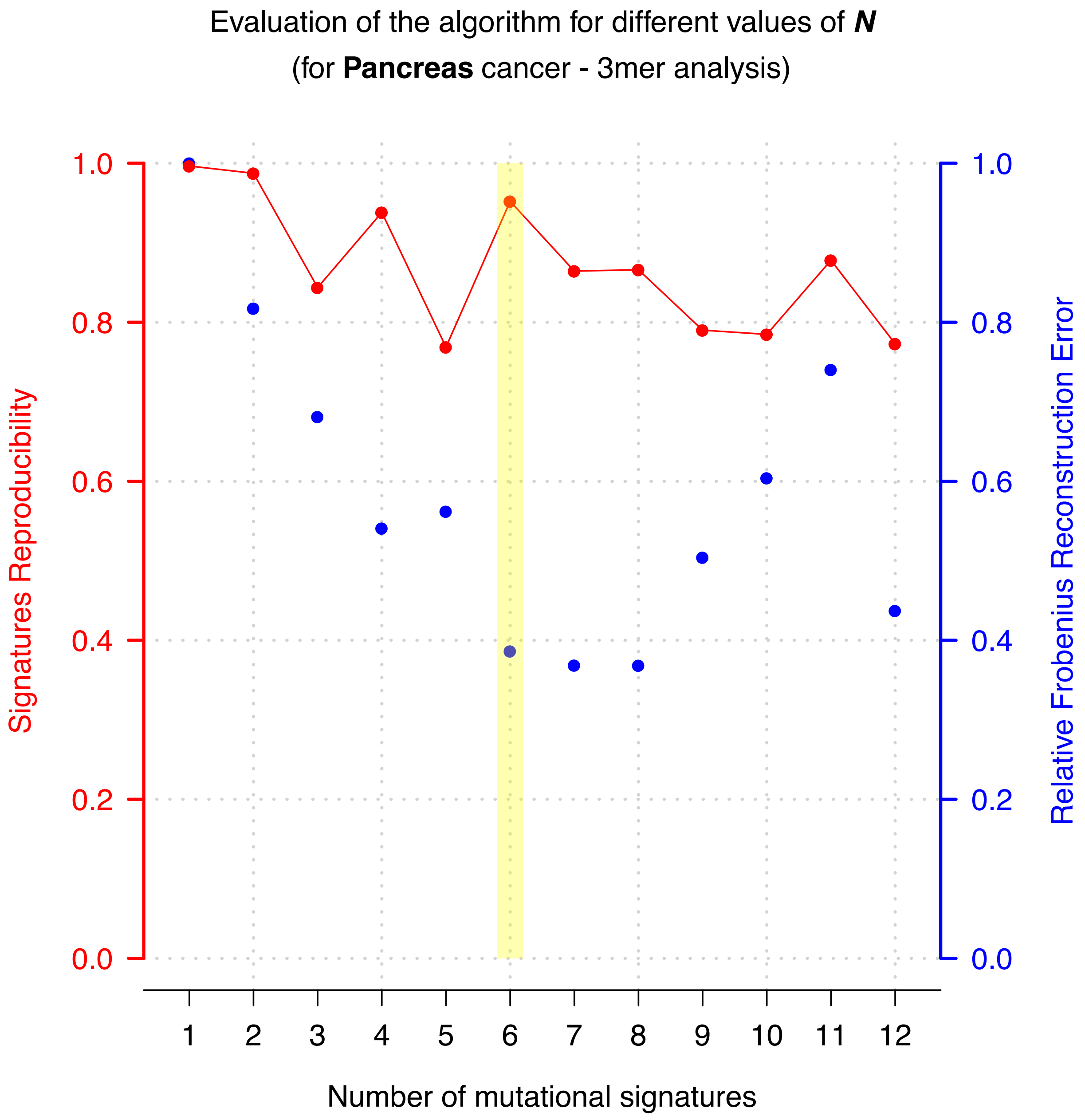

Evaluation of the algorithm for different values of ***N***
(for **Pancreas** cancer - 3mer analysis)
1.0
0.8
0.6
0.4
0.2
0.0
Signatures Reproducibility
Relative Frobenius Reconstruction Error
1 2 3 4 5 6 7 8 9 10 11 12
Number of mutational signatures

Figure S6

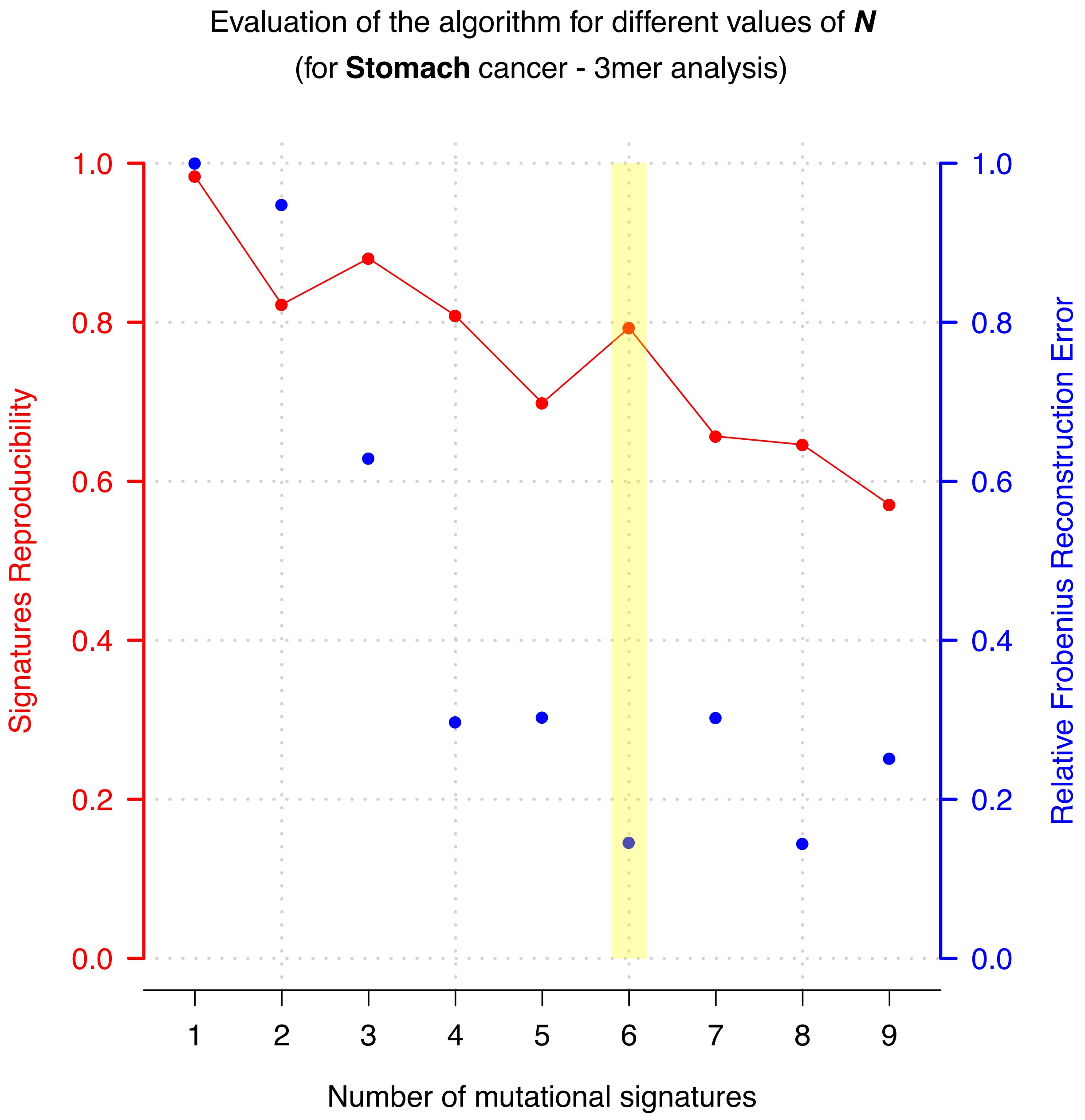

Evaluation of the algorithm for different values of N
(for Stomach cancer - 3mer analysis)
1.0
0.8
0.6
0.4
0.2
0.0
Signatures Reproducibility
Relative Frobenius Reconstruction Error
1
2
3
4
5
6
7
8
9
Number of mutational signatures

Figure S7

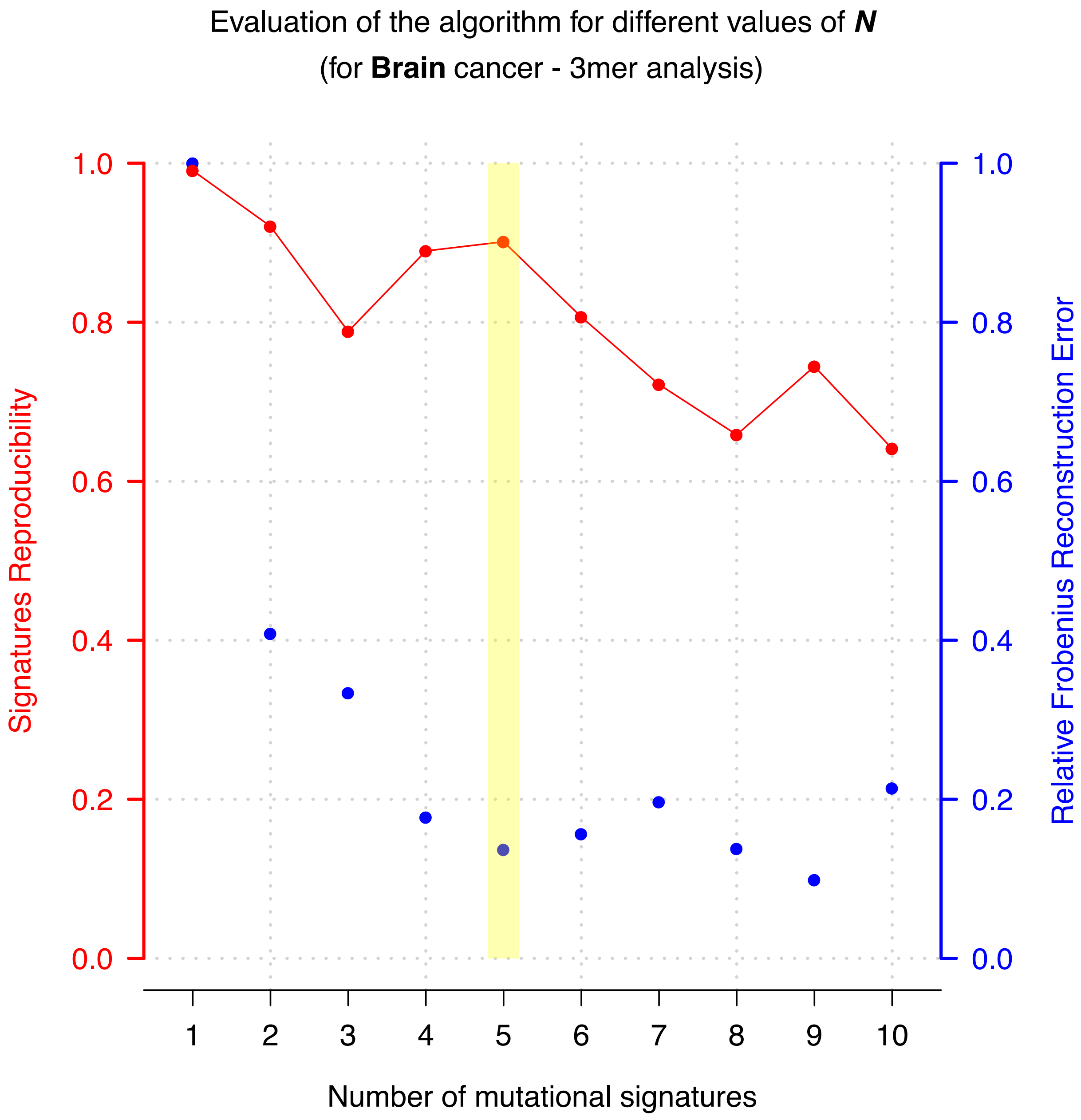
Evaluation of the algorithm for different values of ***N***
(for **Brain** cancer - 3mer analysis)
Signatures Reproducibility
Relative Frobenius Reconstruction Error
1.0
0.8
0.6
0.4
0.2
0.0
1
2
3
4
5
6
7
8
9
10
Number of mutational signatures

Figure S8

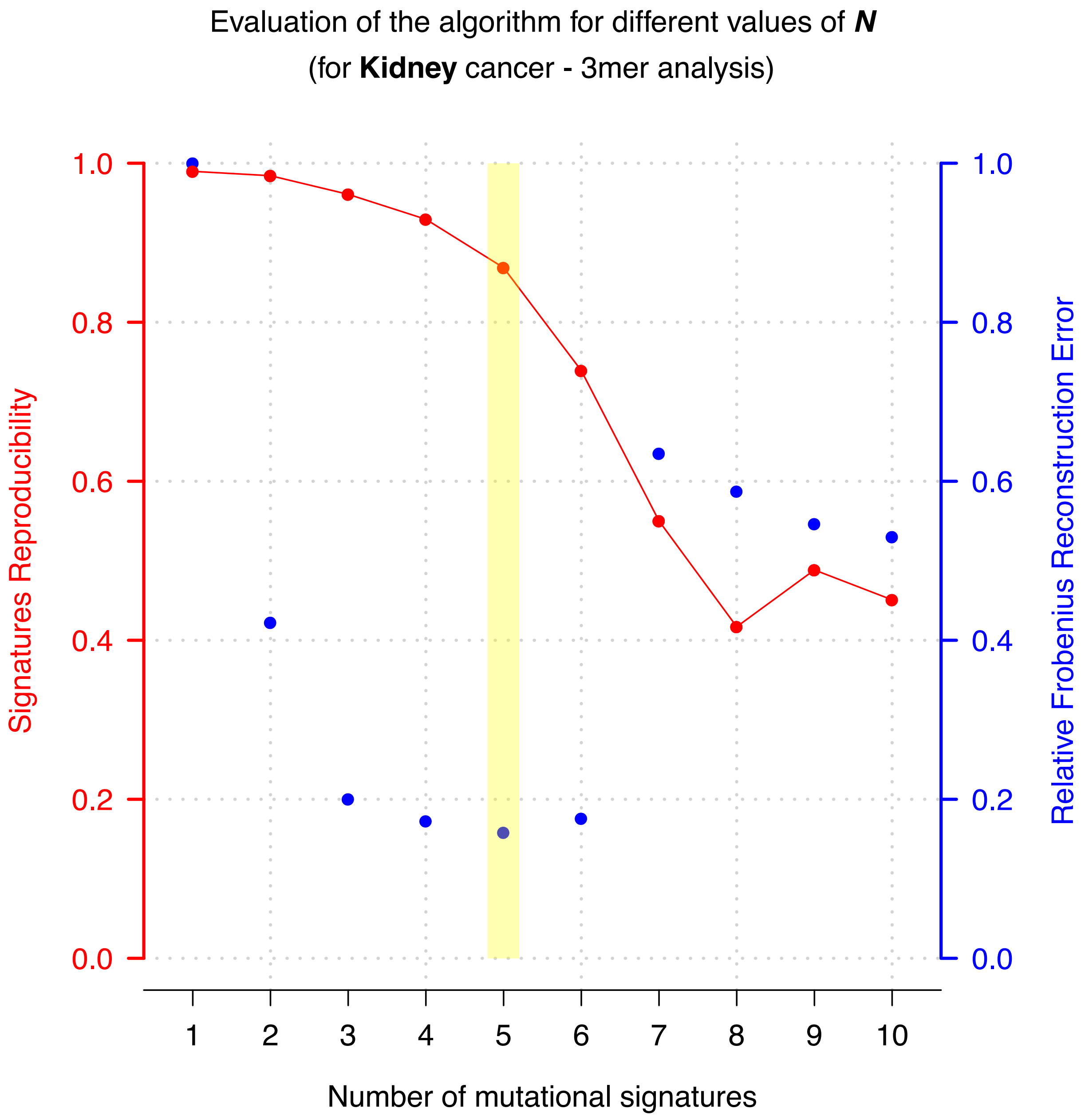
Evaluation of the algorithm for different values of N
(for Kidney cancer - 3mer analysis)
Signatures Reproducibility
Relative Frobenius Reconstruction Error
Number of mutational signatures
0.0
0.2
0.4
0.6
0.8
1.0
1
2
3
4
5
6
7
8
9
10

Figure S9

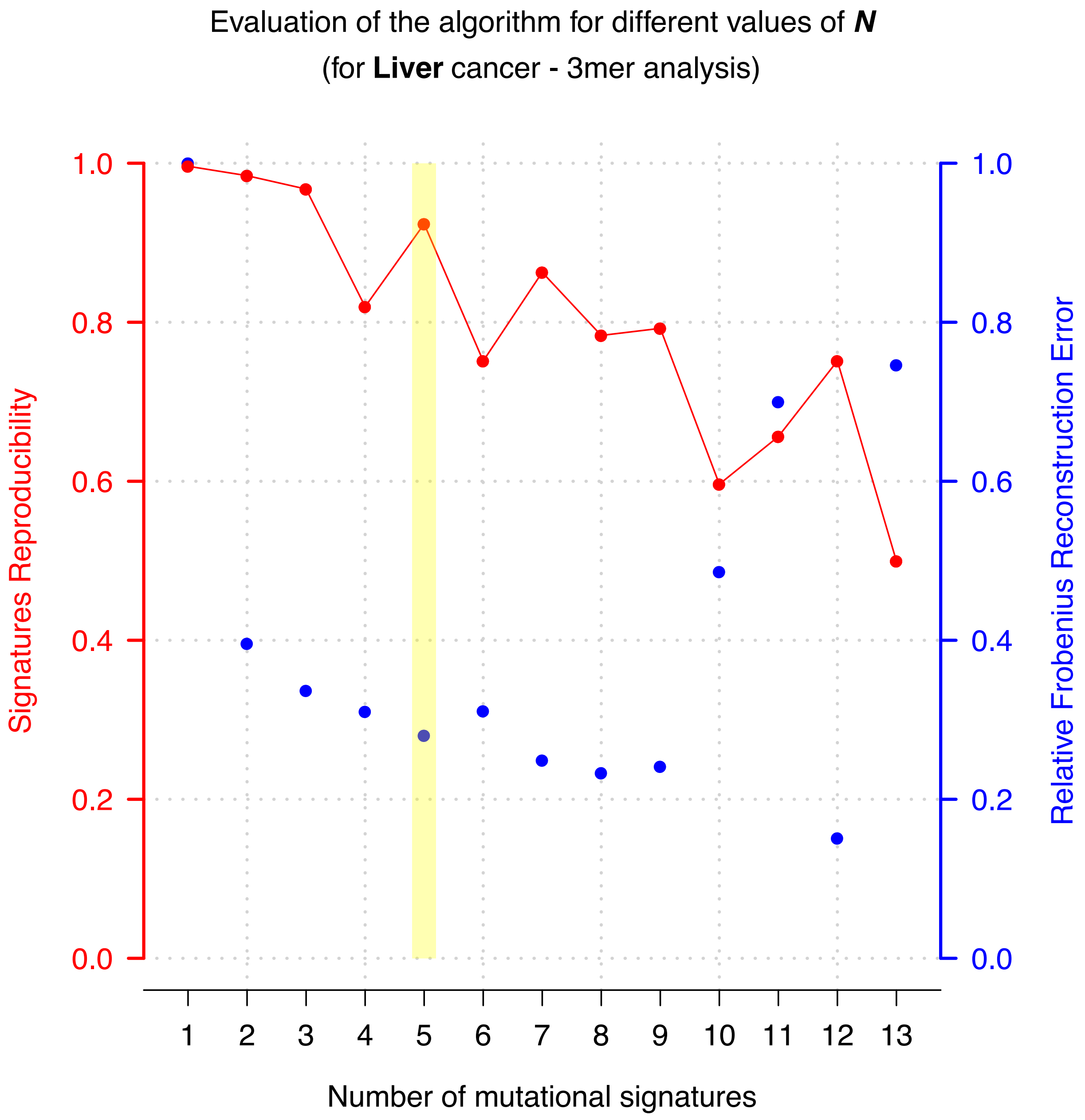
Evaluation of the algorithm for different values of ***N***
(for **Liver** cancer - 3mer analysis)
Signatures Reproducibility
Relative Frobenius Reconstruction Error
1.0
0.8
0.6
0.4
0.2
0.0
1
2
3
4
5
6
7
8
9
10
11
12
13
Number of mutational signatures

Figure S10

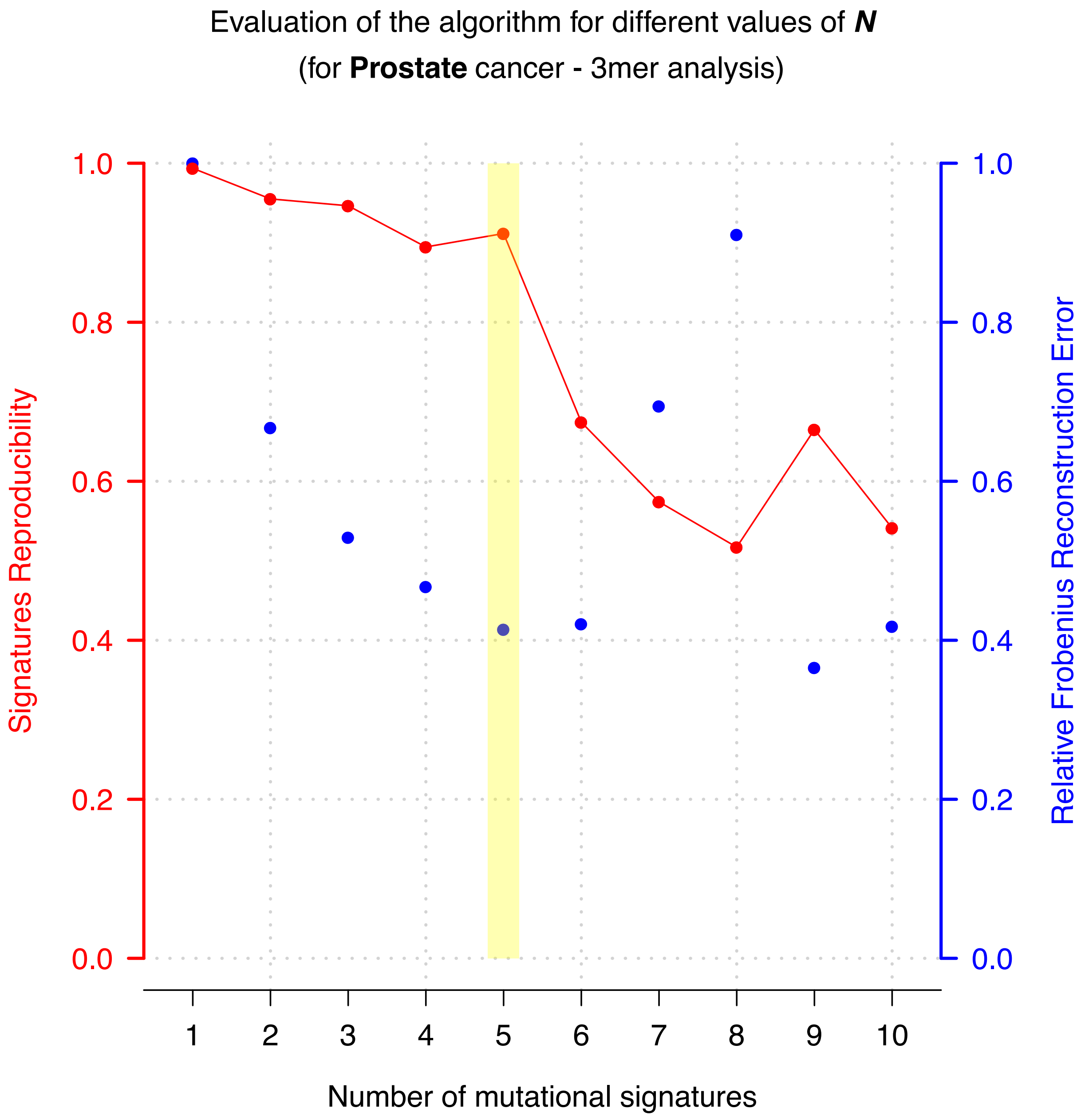
Evaluation of the algorithm for different values of N
(for Prostate cancer - 3mer analysis)
Signatures Reproducibility
Relative Frobenius Reconstruction Error
1.0
0.8
0.6
0.4
0.2
0.0
1
2
3
4
5
6
7
8
9
10
Number of mutational signatures

Figure S11

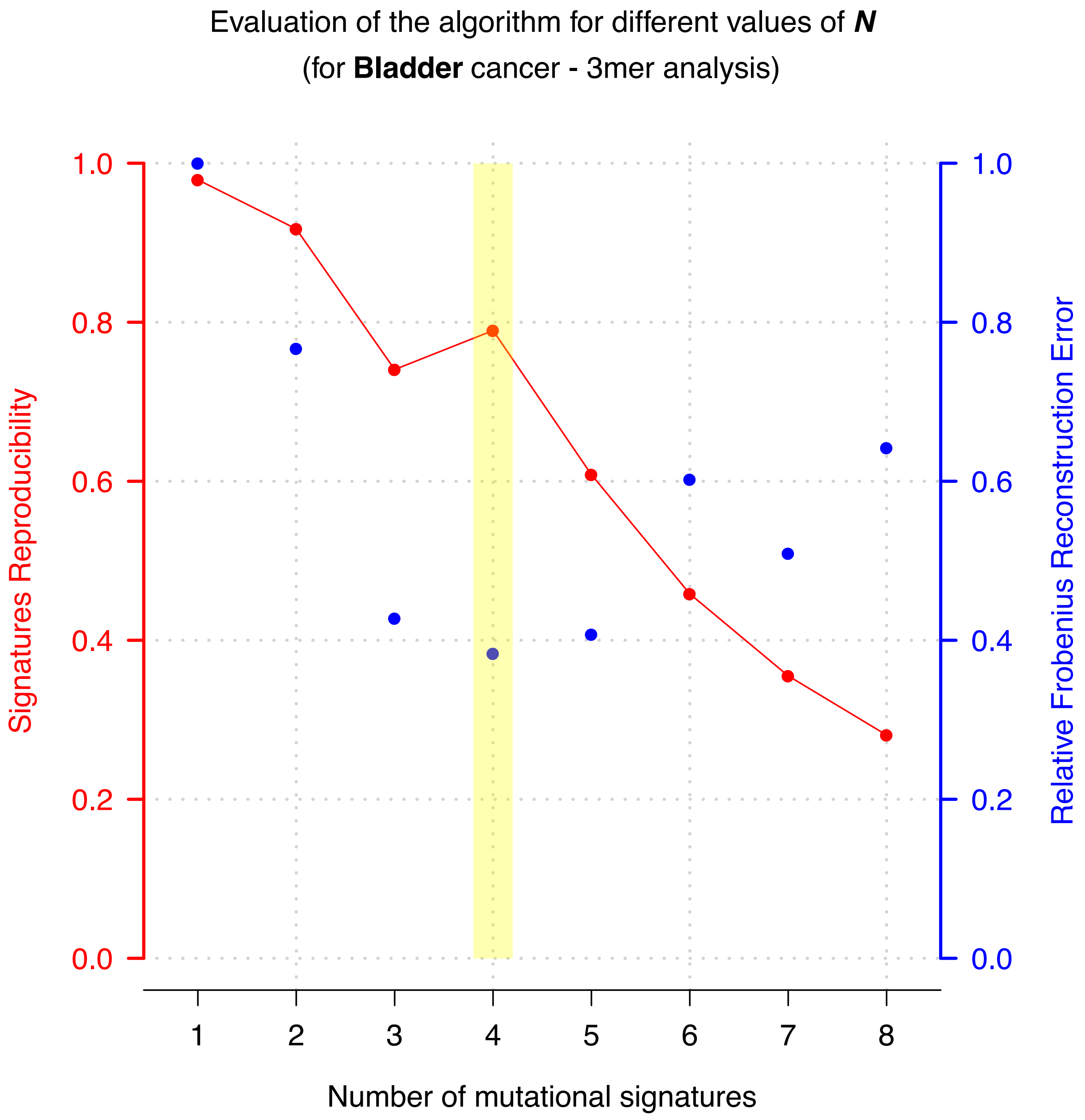
Evaluation of the algorithm for different values of N
(for Bladder cancer - 3mer analysis)
Signatures Reproducibility
Relative Frobenius Reconstruction Error
1.0
0.8
0.6
0.4
0.2
0.0
1
2
3
4
5
6
7
8
Number of mutational signatures

Figure S12

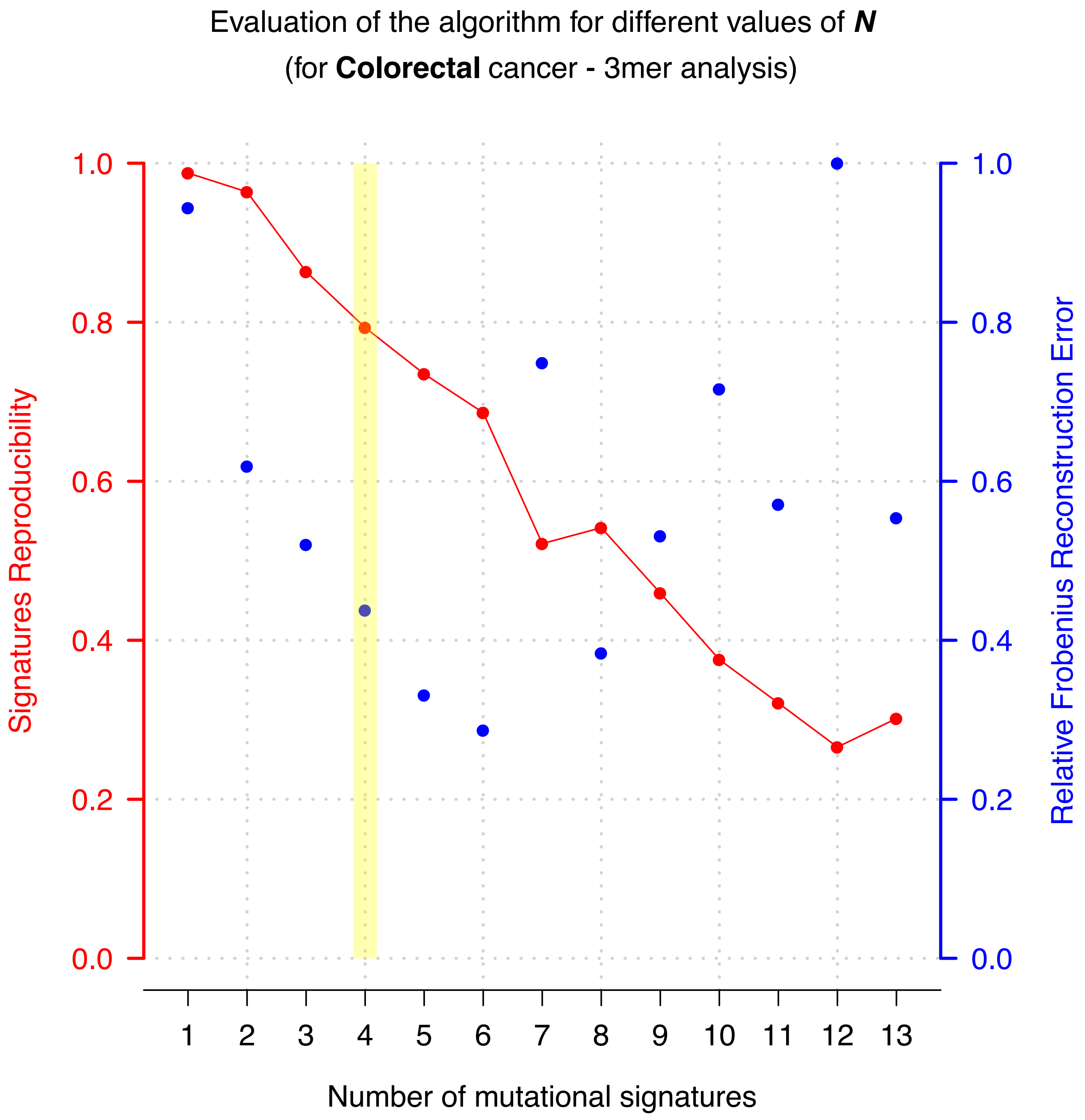

Evaluation of the algorithm for different values of ***N***
(for **Colorectal** cancer - 3mer analysis)
Signatures Reproducibility
Relative Frobenius Reconstruction Error
0.0
0.2
0.4
0.6
0.8
1.0
1
2
3
4
5
6
7
8
9
10
11
12
13
Number of mutational signatures

Figure S13

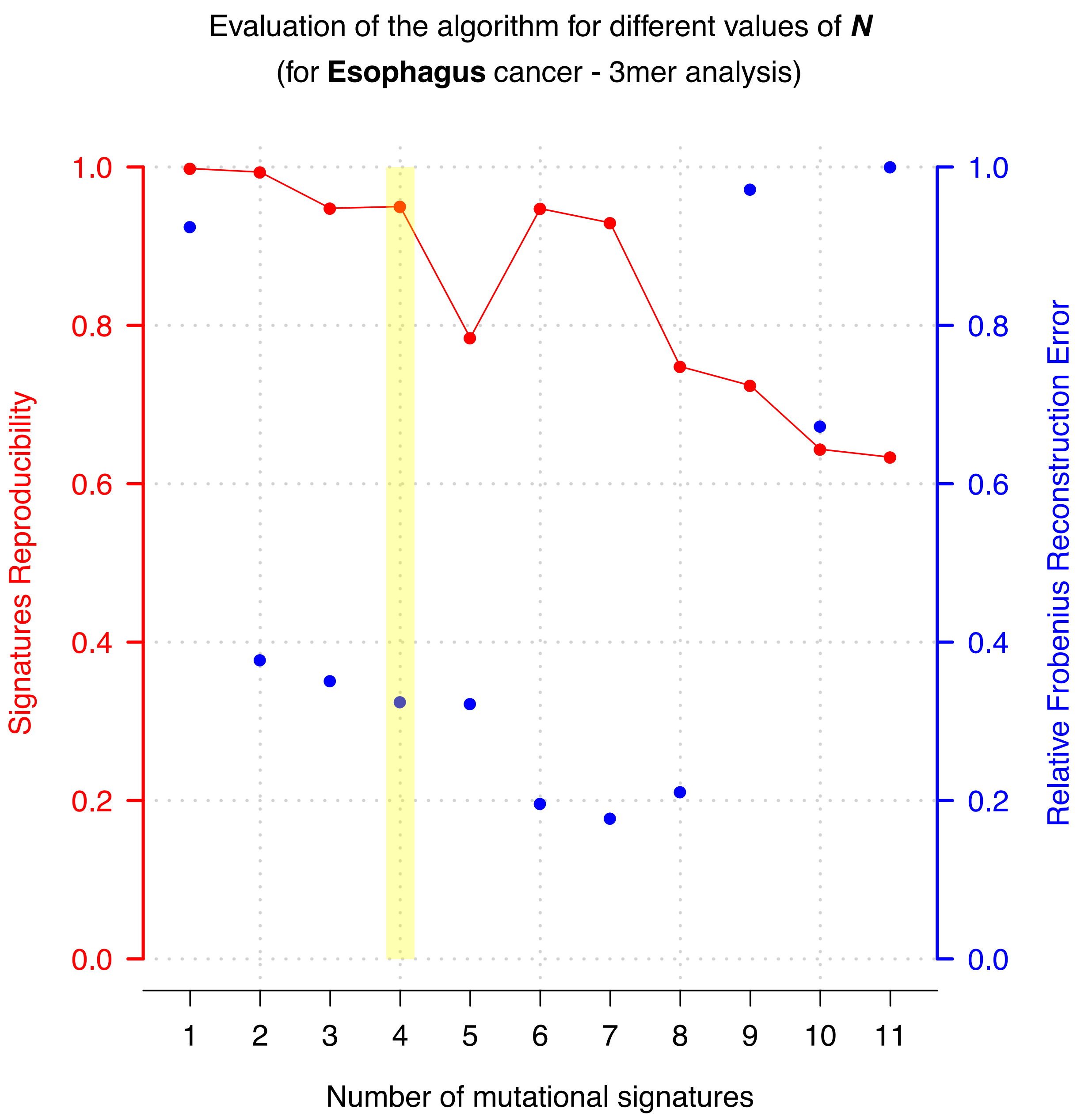

Evaluation of the algorithm for different values of ***N***
(for **Esophagus** cancer - 3mer analysis)
Signatures Reproducibility
Relative Frobenius Reconstruction Error
Number of mutational signatures
0.0
0.2
0.4
0.6
0.8
1.0
1
2
3
4
5
6
7
8
9
10
11

Figure S14

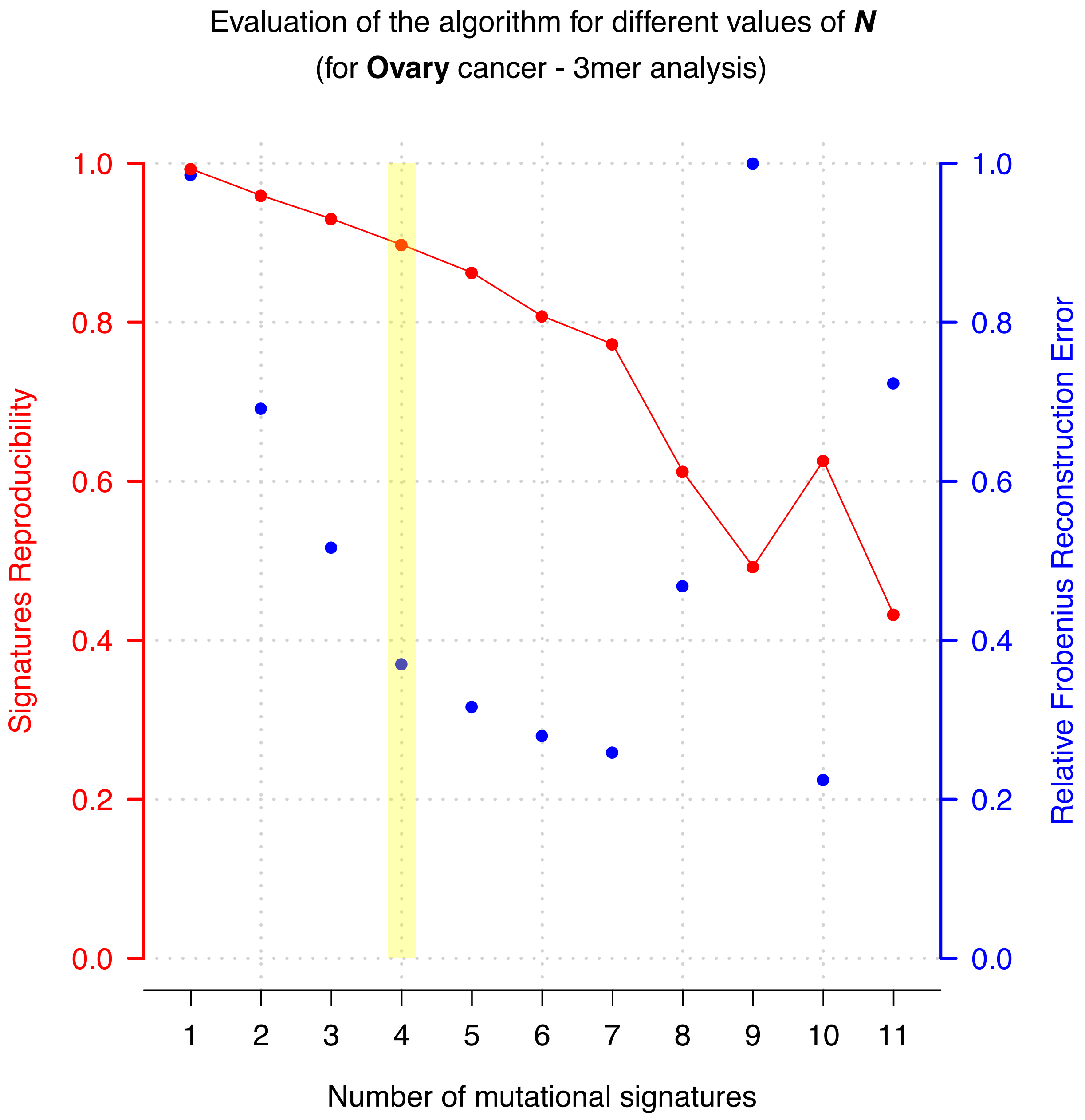

Evaluation of the algorithm for different values of ***N***
(for **Ovary** cancer - 3mer analysis)
Signatures Reproducibility
Relative Frobenius Reconstruction Error
1.0
0.8
0.6
0.4
0.2
0.0
1
2
3
4
5
6
7
8
9
10
11
Number of mutational signatures

Figure S15

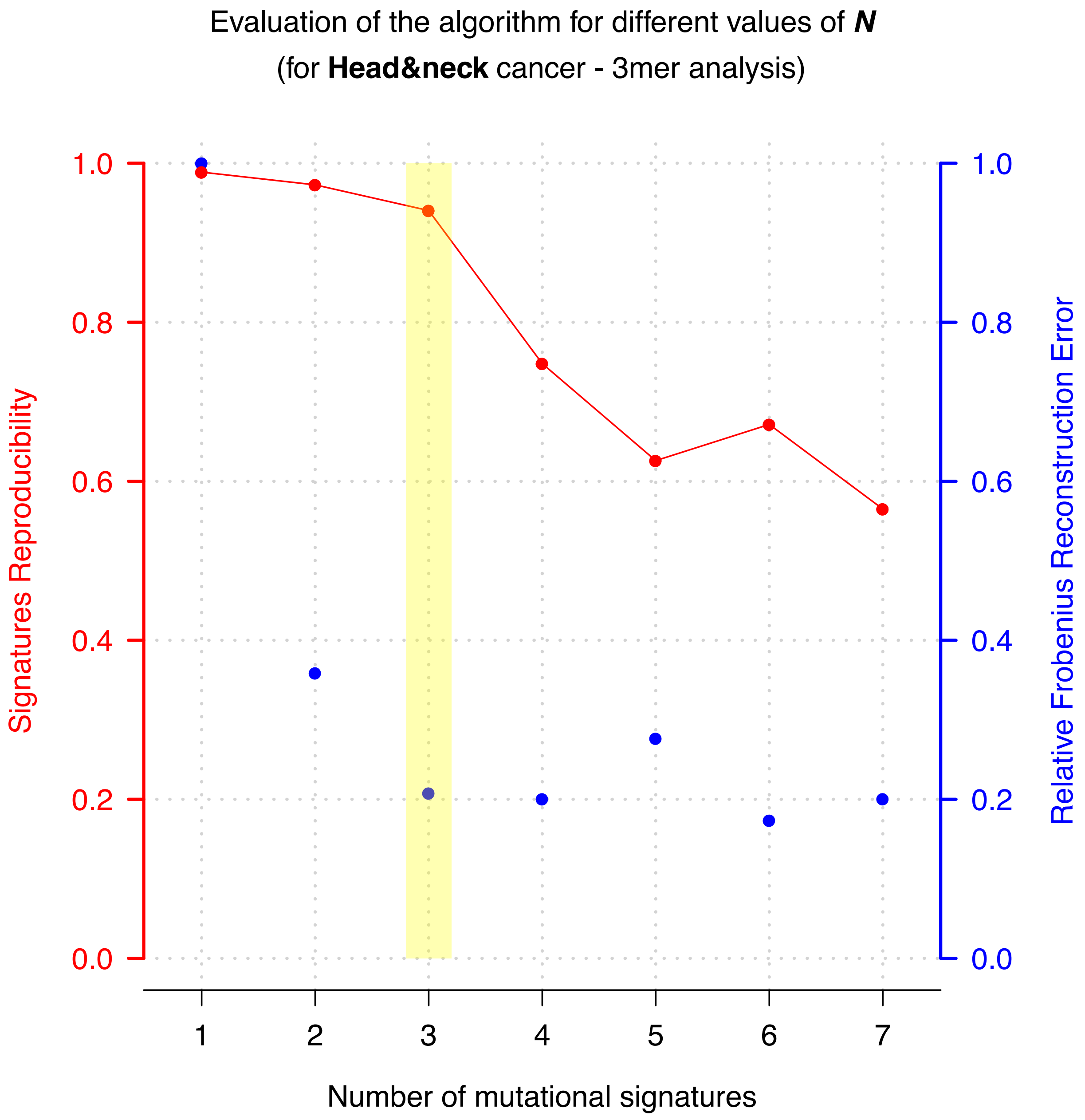

Evaluation of the algorithm for different values of ***N***
(for **Head&neck** cancer - 3mer analysis)
Signatures Reproducibility
Relative Frobenius Reconstruction Error
Number of mutational signatures
0.0
0.2
0.4
0.6
0.8
1.0
1
2
3
4
5
6
7

Figure S16

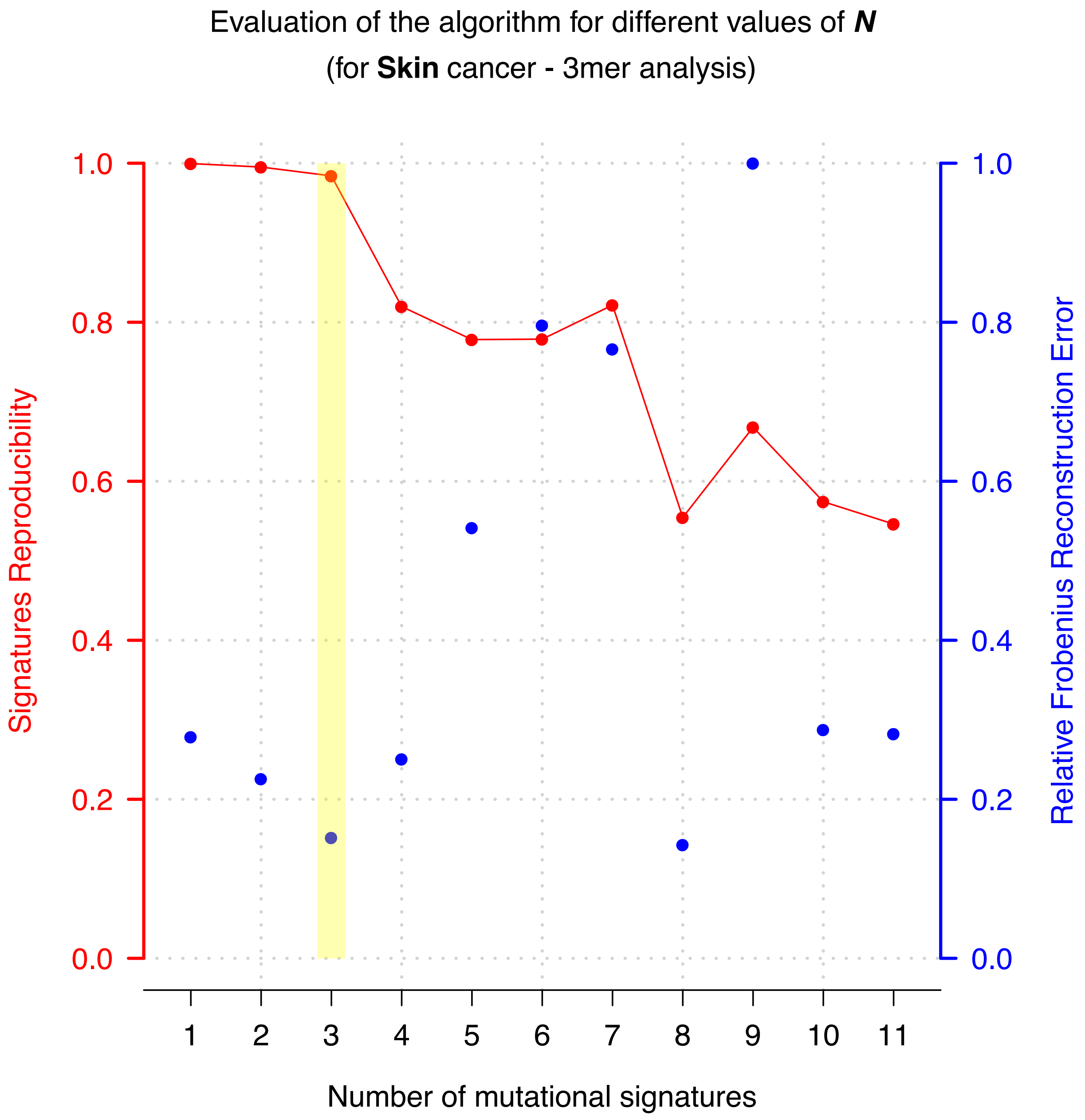

Evaluation of the algorithm for different values of ***N***
(for **Skin** cancer - 3mer analysis)
1.0
0.8
0.6
0.4
0.2
0.0
Signatures Reproducibility
1.0
0.8
0.6
0.4
0.2
0.0
Relative Frobenius Reconstruction Error
1
2
3
4
5
6
7
8
9
10
11
Number of mutational signatures

Figure S17

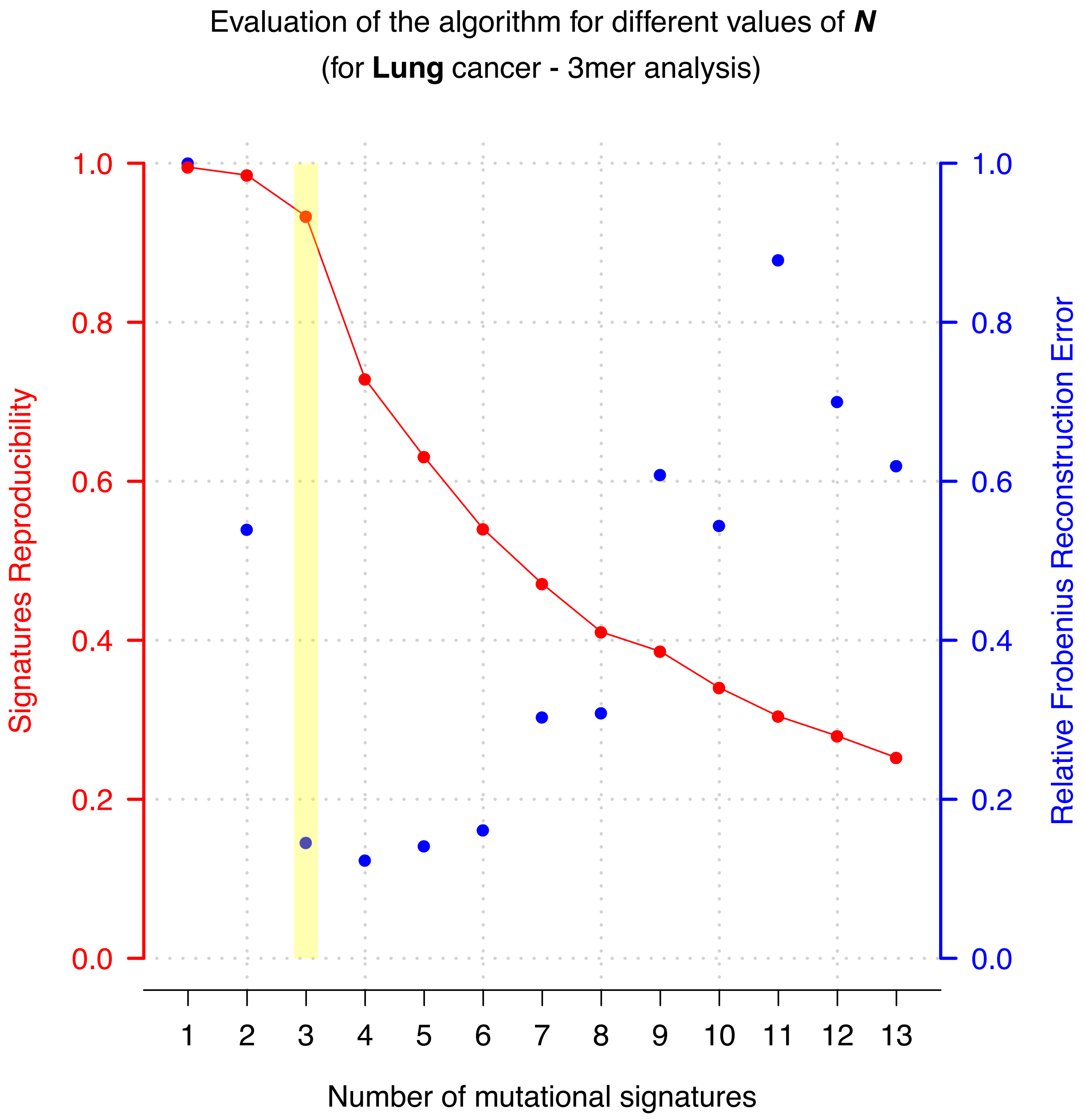

Evaluation of the algorithm for different values of ***N***
(for **Lung** cancer - 3mer analysis)
Signatures Reproducibility
Relative Frobenius Reconstruction Error
0.0
0.2
0.4
0.6
0.8
1.0
1
2
3
4
5
6
7
8
9
10
11
12
13
Number of mutational signatures

Figure S18

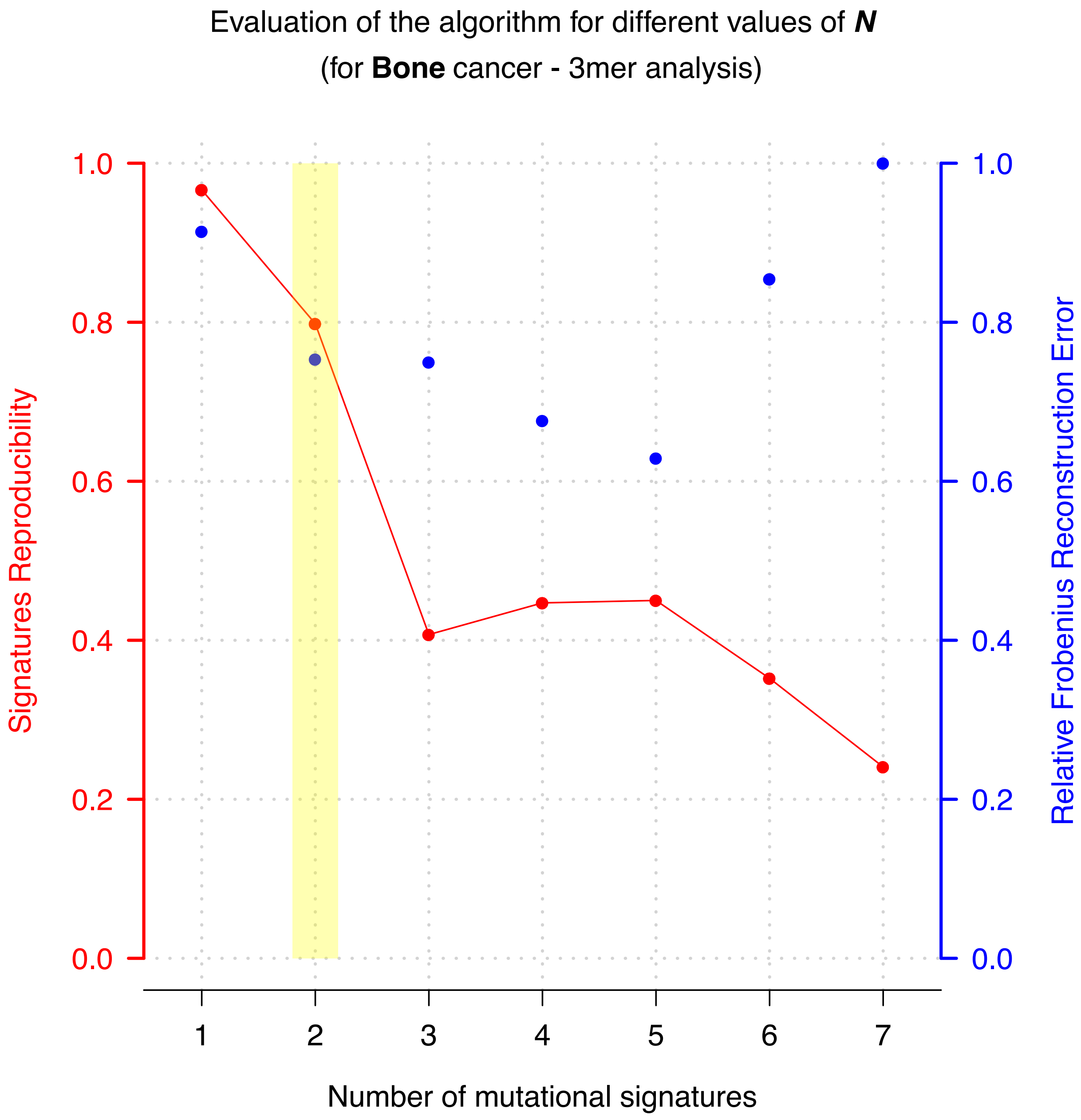
Evaluation of the algorithm for different values of ***N***
(for **Bone** cancer - 3mer analysis)
Signatures Reproducibility
Relative Frobenius Reconstruction Error
Number of mutational signatures
0.0
0.2
0.4
0.6
0.8
1.0
1
2
3
4
5
6
7

Figure S19

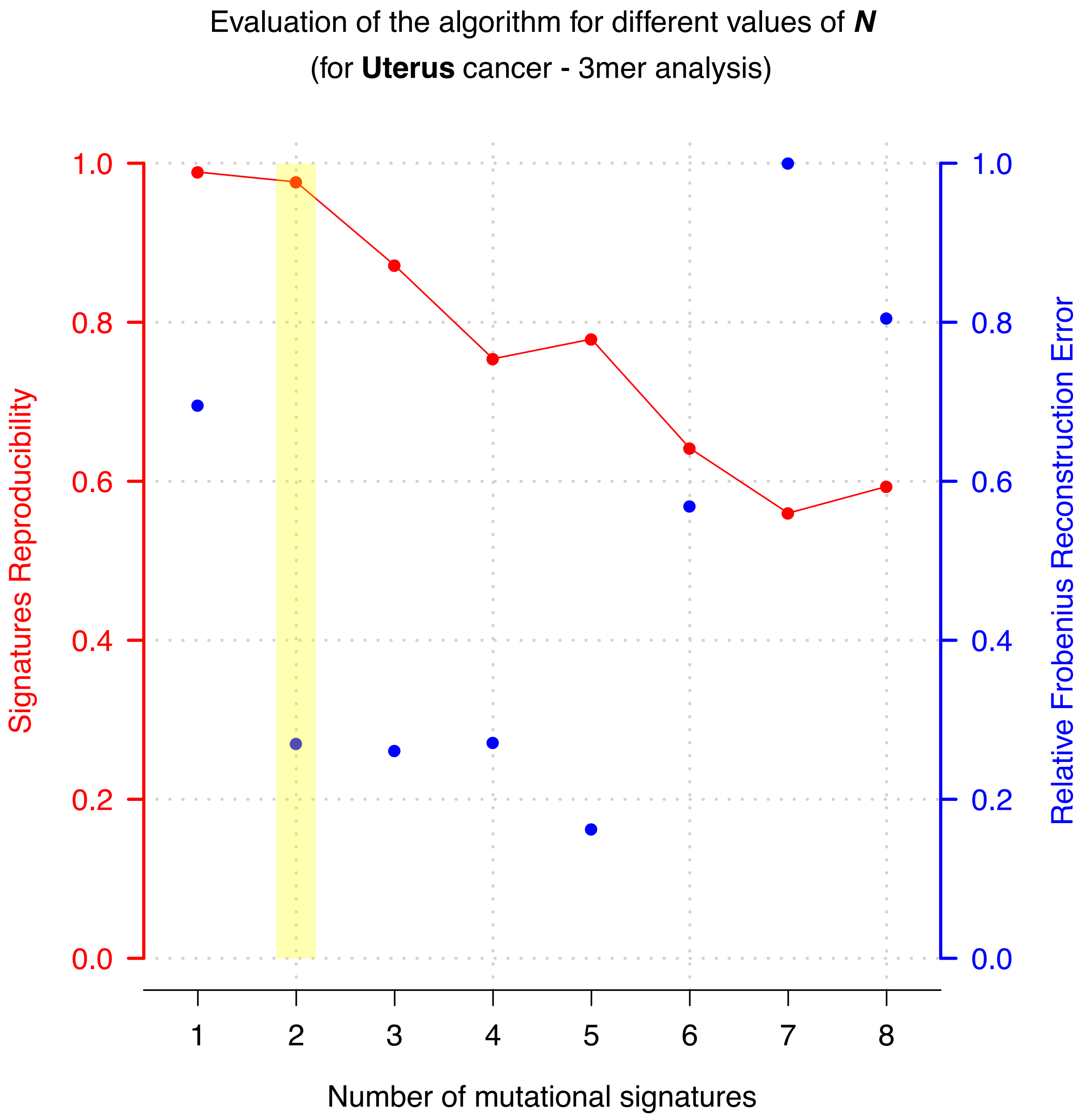
Evaluation of the algorithm for different values of ***N***
(for **Uterus** cancer - 3mer analysis)
Signatures Reproducibility
Relative Frobenius Reconstruction Error
Number of mutational signatures
0.0
0.2
0.4
0.6
0.8
1.0
1
2
3
4
5
6
7
8

Figure S20

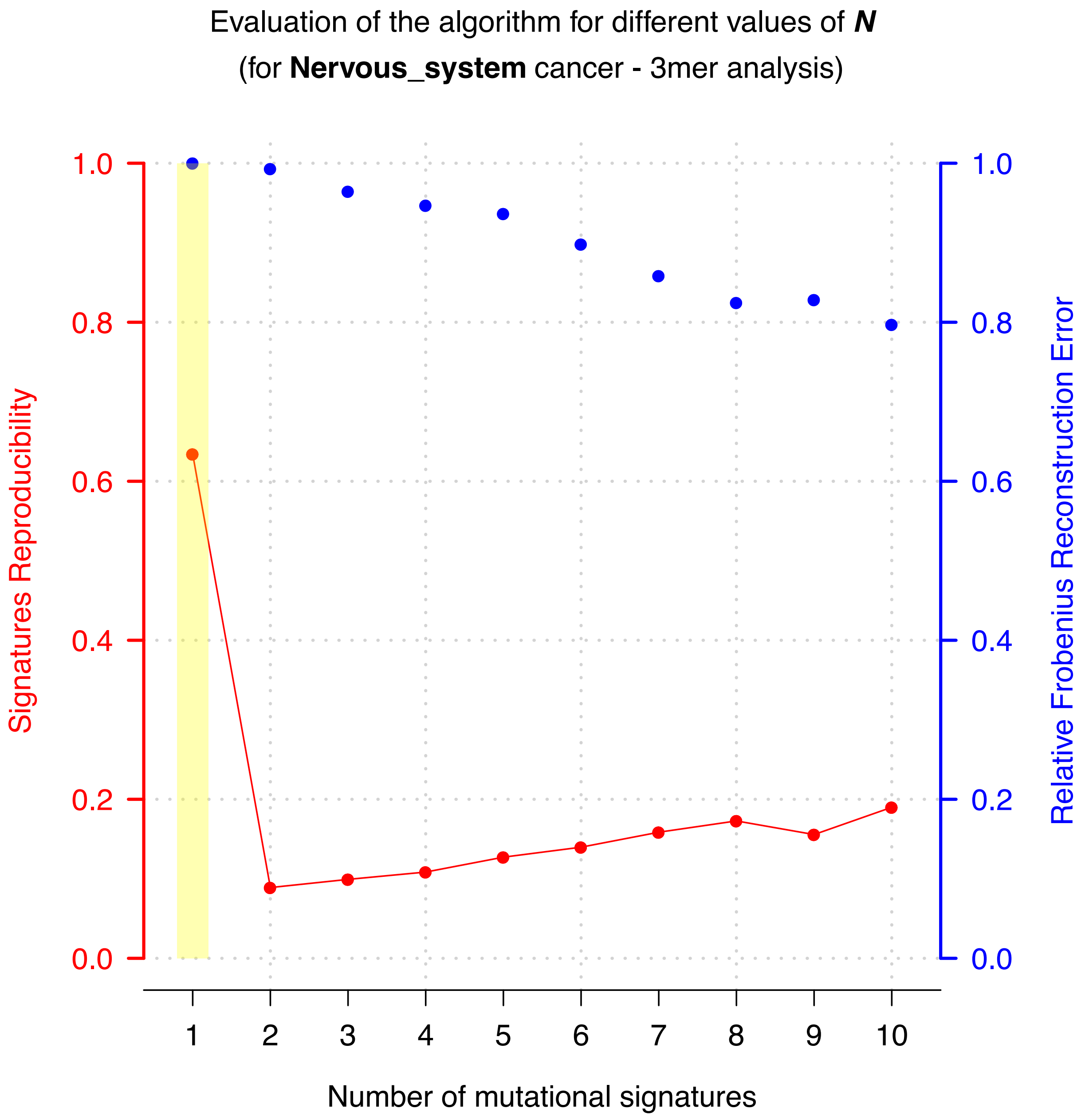

Evaluation of the algorithm for different values of ***N***
(for **Nervous_system** cancer - 3mer analysis)
1.0
0.8
0.6
0.4
0.2
0.0
Signatures Reproducibility
Relative Frobenius Reconstruction Error
1
2
3
4
5
6
7
8
9
10
Number of mutational signatures

Figure S21

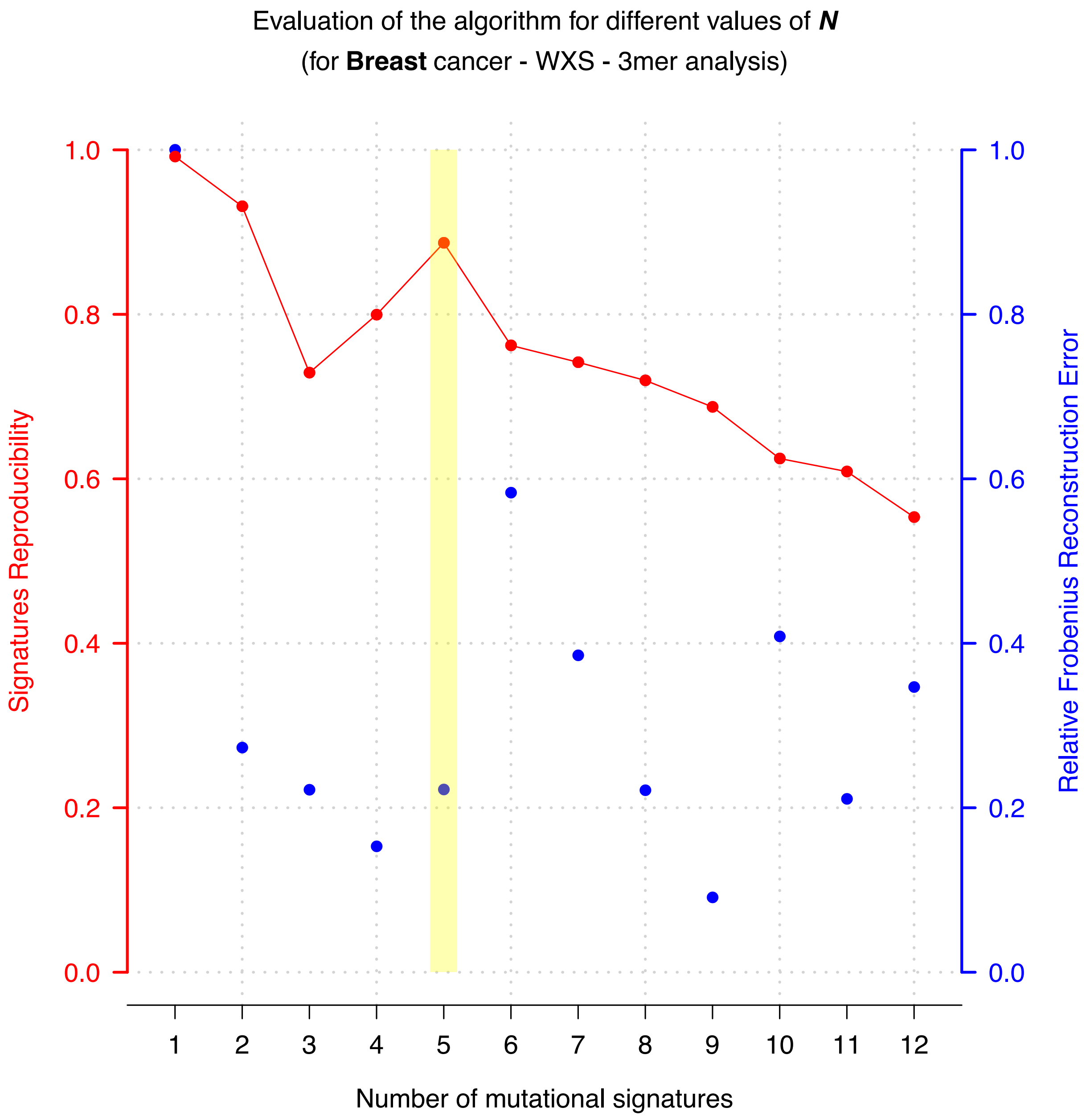

Evaluation of the algorithm for different values of ***N***
(for **Breast** cancer - WXS - 3mer analysis)
Signatures Reproducibility
Relative Frobenius Reconstruction Error
Number of mutational signatures
1.0
0.8
0.6
0.4
0.2
0.0
1
2
3
4
5
6
7
8
9
10
11
12

Figure S22

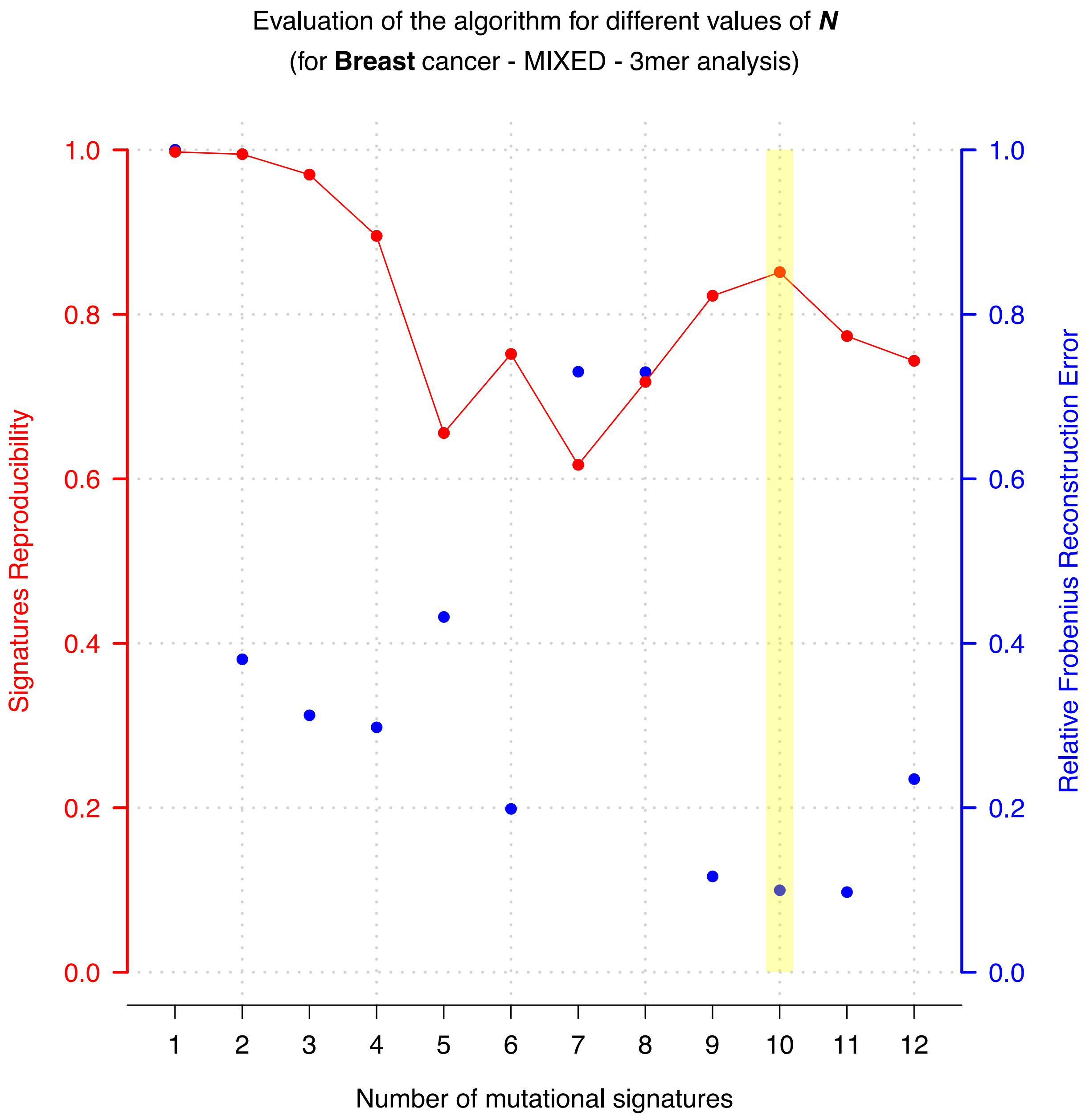

Evaluation of the algorithm for different values of ***N***
(for **Breast** cancer - MIXED - 3mer analysis)
Signatures Reproducibility
Relative Frobenius Reconstruction Error
1.0
0.8
0.6
0.4
0.2
0.0
1
2
3
4
5
6
7
8
9
10
11
12
Number of mutational signatures

Figure S23

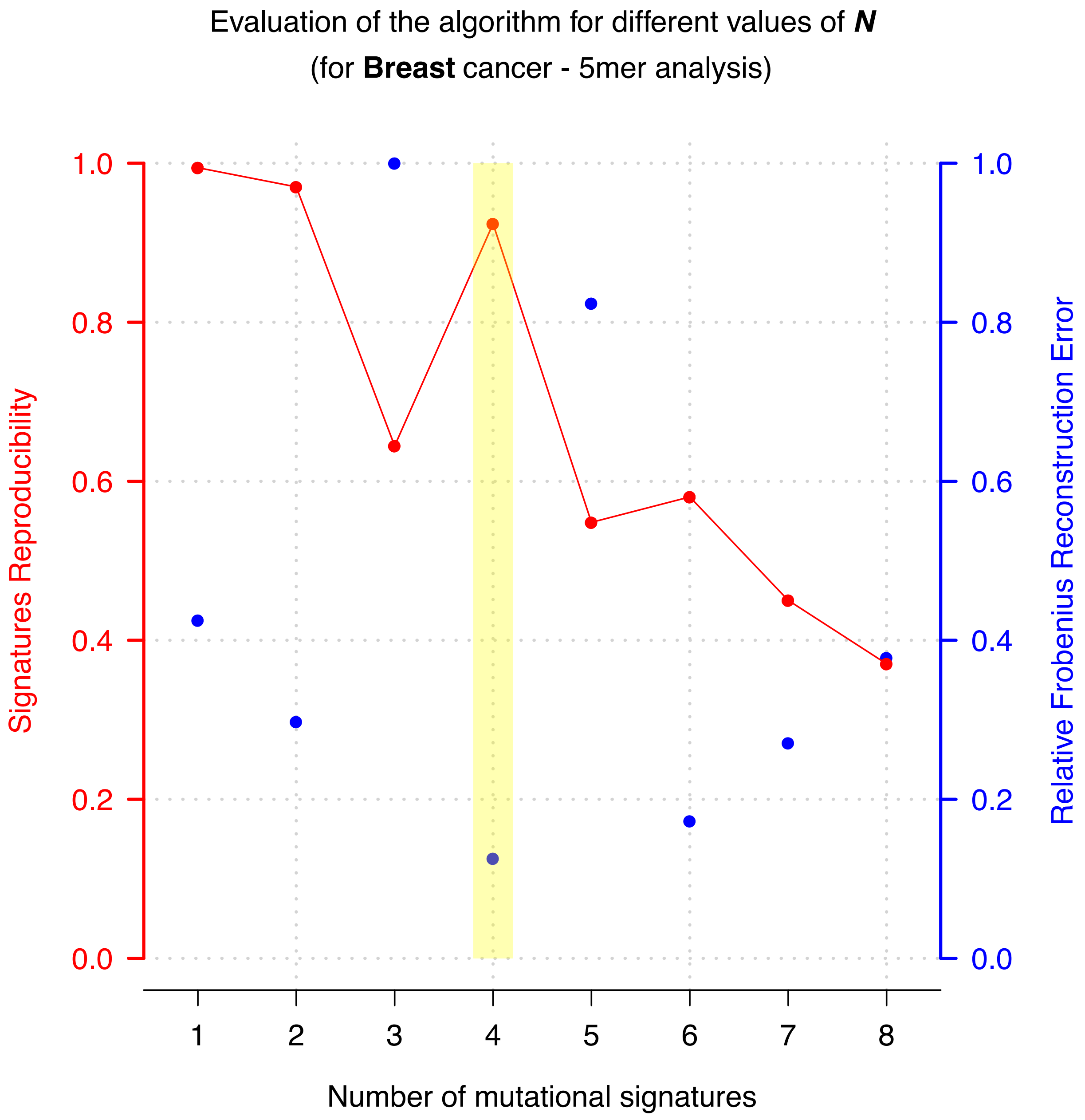
Evaluation of the algorithm for different values of N
(for Breast cancer - 5mer analysis)
Signatures Reproducibility
Relative Frobenius Reconstruction Error
Number of mutational signatures
1.0
0.8
0.6
0.4
0.2
0.0
1
2
3
4
5
6
7
8

Figure S24

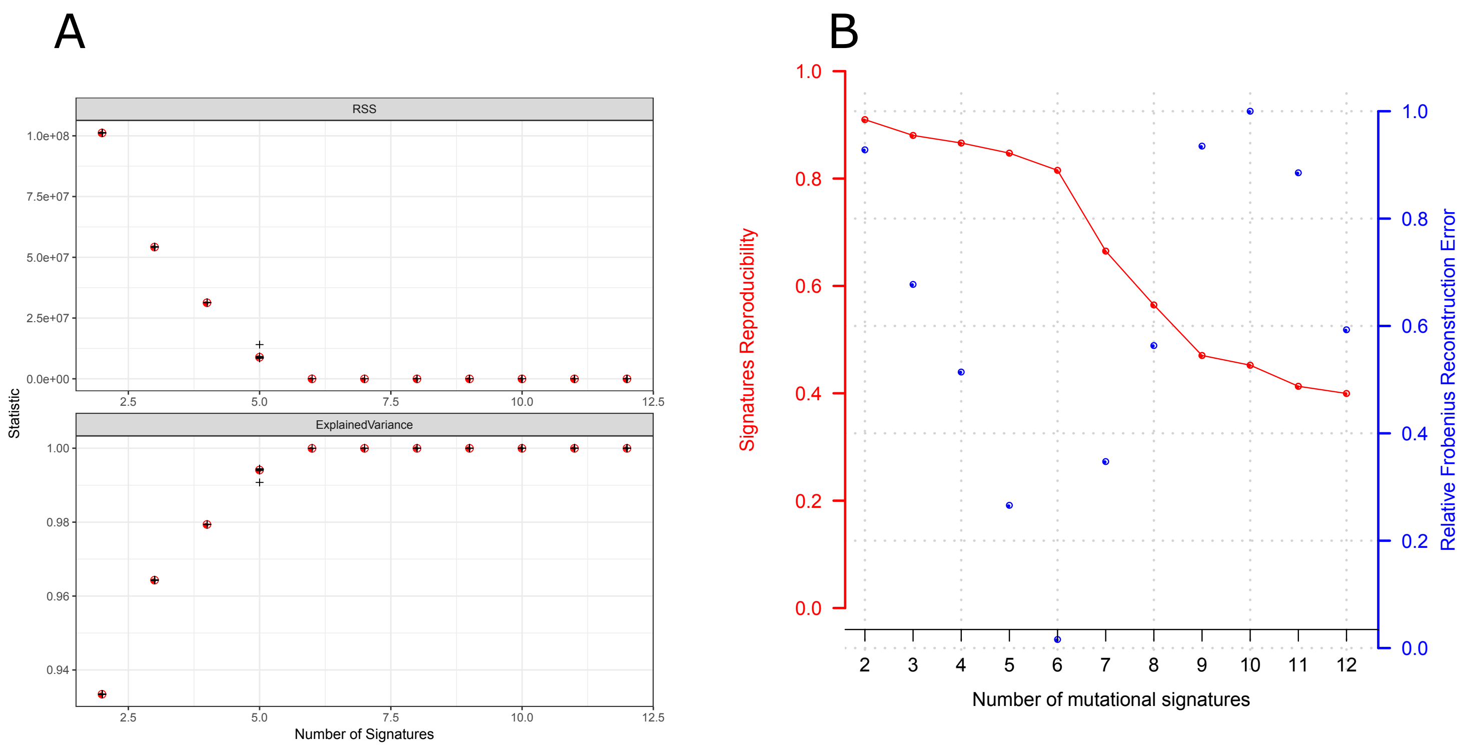
A
RSS
1.0e+08
7.5e+07
5.0e+07
2.5e+07
0.0e+00
Statistic
ExplainedVariance
1.00
0.98
0.96
0.94
2.5
5.0
7.5
10.0
12.5
Number of Signatures
B
Signatures Reproducibility
1.0
0.8
0.6
0.4
0.2
0.0
Relative Frobenius Reconstruction Error
2
3
4
5
6
7
8
9
10
11
12
Number of mutational signatures

Figure S25

A

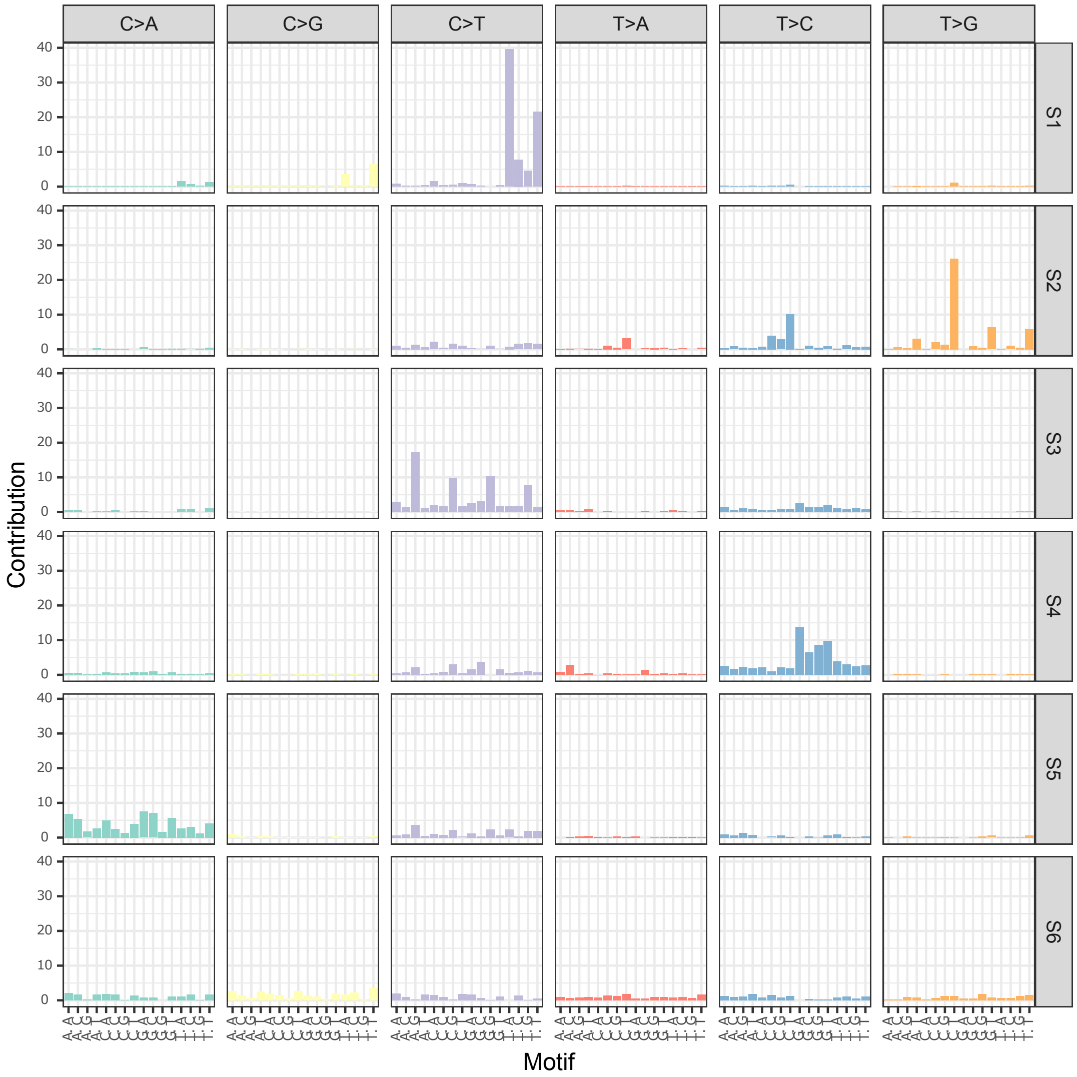

B

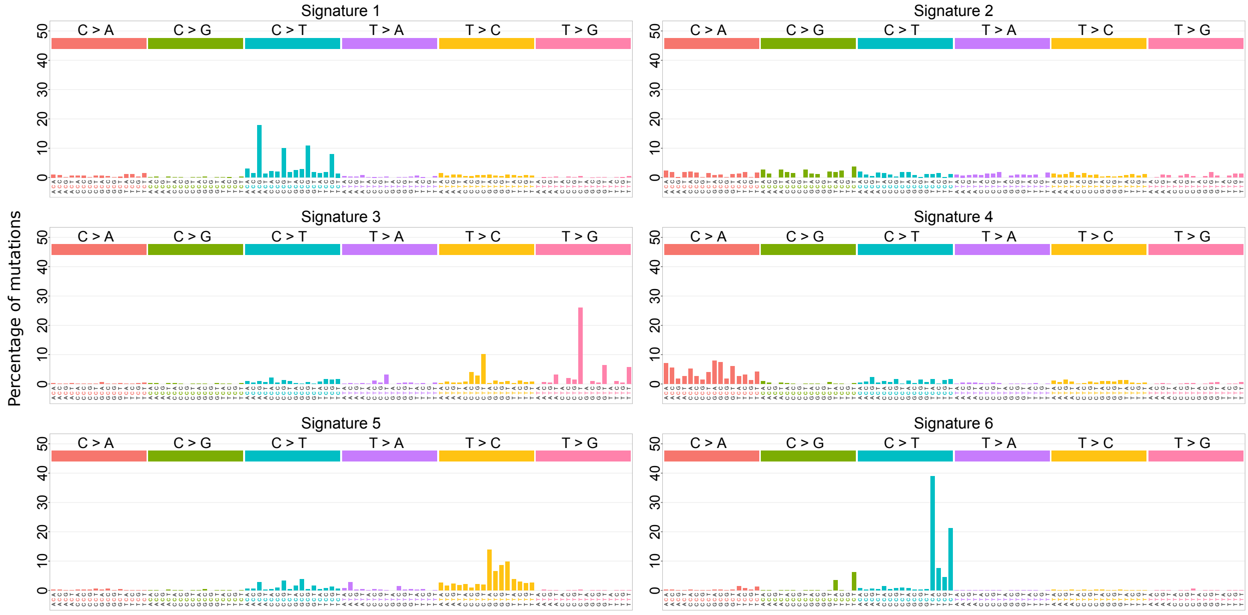

Figure S26

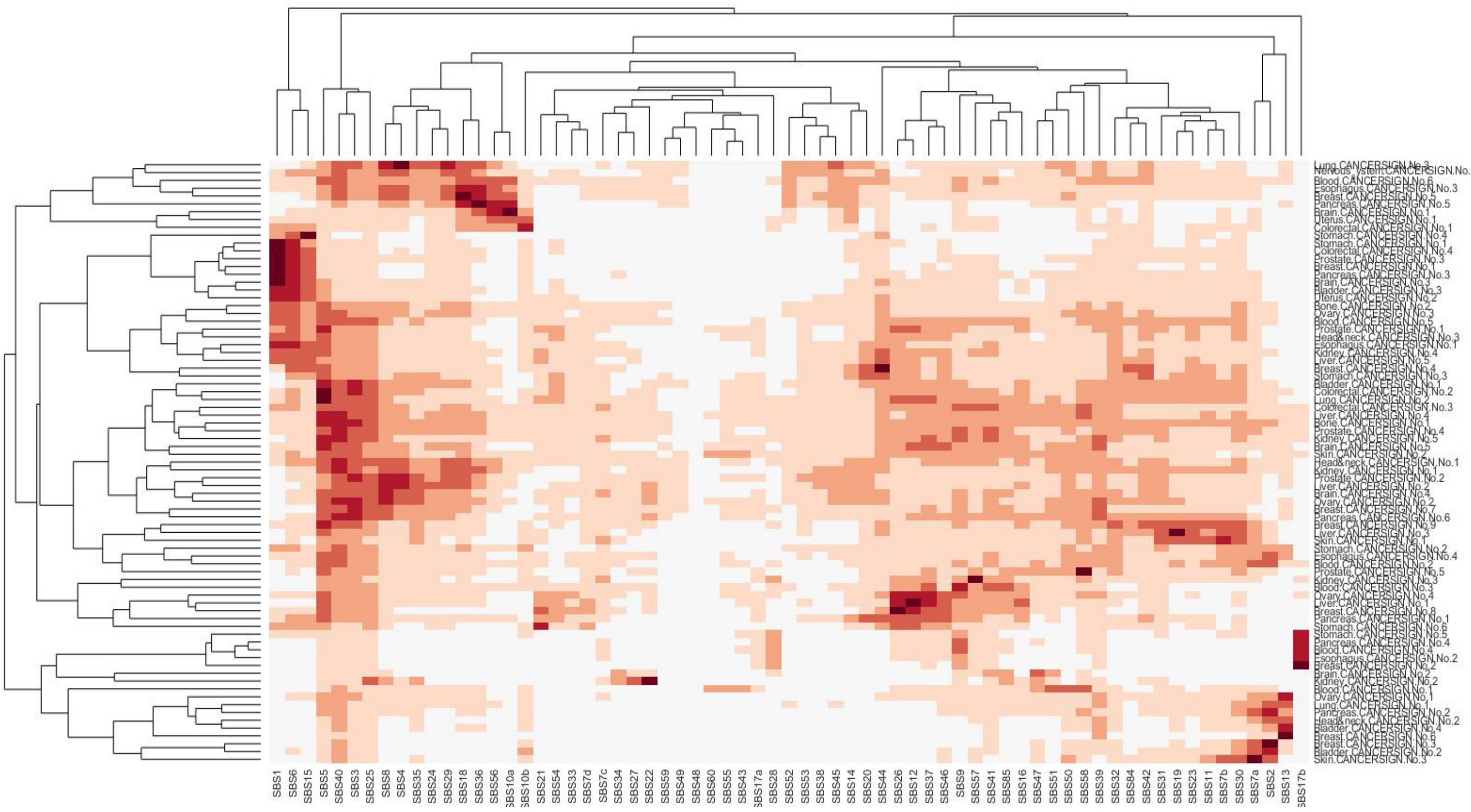

CANCERSIGN User Manual

1. Requirements

— **R** is required to be installed and the command “**Rscript**” must be available.
— The following packages must be installed for **R**:

- BSgenome.Hsapiens.UCSC.hg19
- data.table
- doParallel
- ggplot2
- configr

2. Initial setup

Download CANCERSIGN.gz and uncompress it using gunzip command:

```
>> gunzip </path/to/CANCERSIGN.gz>
```

where </path/to/CANCERSIGN.gz> is the path to **CANCERSIGN.gz** file.

Then run the following commands to make the command “**cancersign**” active (executable):

```
>> cd </path/to/CANCERSIGN>
>> chmod +x cancersign
>> export PATH=$PATH:</path/to/CANCERSIGN>
```

where </path/to/CANCERSIGN> is the path to **CANCERSIGN** folder. For the last command, this path must be ***absolute*** (not relative).

3. Run CANCERSIGN

Before running CANCERSIGN, the user has to provide a simple text file which contains the custom configurations including the path to the input data, the path to the output directory where the user wants the results to be stored in and the analysis parameters (guidelines for input data format and writing the configuration file are provided in the subsequent sections). Assuming that the configurations are written in a file named config.txt, CANCERSIGN starts the analyses with the following command:

```
>> cancersign --config </path/to/config.txt>
```

where </path/to/config.txt> is the path to the configuration file.

4. Input data for CANCERSIGN

The input data must be a tab-delimited file with the following fields:

1. **sample_id** - The ID of the sample.
2. **chromosome** - The name of the chromosome (chr1, chr2, chr3, …, chrX, chrY or chrM).
3. **position** - The position of the mutation in the chromosome.
4. **reference** - The nucleotide at the corresponding location on the reference genome.
5. **mutated_to** - The mutated nucleotide at the corresponding location in the sample.

5. Creating configuration file

The configuration file is a simple text file with lines defining the settings required for the analyses. The first two lines must define the path to the input file and the path to the output directory where the user wants the results of analyses to be stored in. These two lines are written as follows:

```
input_file = </path/to/input_data_file>
output_dir = </path/to/output_dir>
```

where </path/to/input_file> and </path/to/output_dir> are paths to the input data file and to the output directory respectively.

The rest of the lines in the configuration file determine the desired types of analyses as well as the parameters for those analyses. In short, one line is written to “enable” each desired analysis and it is followed by other lines which set the corresponding parameters for that analysis. The following sections explain the configurations for each analysis type.

5.1. Infer 3-mer mutational signatures

To enable this analysis, write the following line in the configuration file:

```
infer_3mer_signatures = yes
```

All parameters for this analysis have initial default values unless the user specifies them with the following lines:

```
N_min_3mer = <a number, default: 1>
```

- The minimum number for testing the number of signatures

```
N_max_3mer = <a number, default: 10>
```

- The maximum number for testing the number of signatures

```
CPU_3mer = <a number, default: 30>
```
- The number of allocated CPU cores for this analysis

```
nmf_iters_3mer = <a number, default: 1e4>
```
- Number of iterations for NMF algorithm in each epoch

```
nmf_conv_3mer = <a number, default: 1e-5>
```
- Convergence threshold for stopping NMF iterations

```
nmf_max_3mer = <a number, default: 5e5>
```
- Maximum number of iterations for NMF algorithm

```
boot_iters_3mer = <a number, default: 30 or number of available CPUs>
```
- Number of bootstrap iterations performed in each epoch

```
boot_conv_3mer = <a number, default: 0.01>
```
- The convergence threshold for stopping bootstrap iterations

```
boot_max_3mer = <a number, default: 600>
```
- Maximum number of bootstrap iterations

The results of this analysis are stored in the output directory in a folder named "infered_3mer_signatures".

5.2. Infer 5-mer mutational signatures

To enable this analysis, write the following line in the configuration file:

```
infer_5mer_signatures = yes
```

The parameter that the user must provide for this analysis is the desired set of 3-mer motifs. Based on this parameter, the tool expands the specified 3-mer motifs to all possible 5-mer motifs (which contain the specified 3-mer motifs) and then infers the 5-mer mutational signatures corresponding to them. This parameter is specified with the following line:

```
selected_3mer_motifs_for_5mer_signatures = <motif-1>, ..., <motif-n>
```
where <motif-i> is a selected 3mer motif in a standard format. An example of this standard format is "**G[C>T]A**" which means C>T mutation with G as left flanking nucleotide and A as the right flanking nucleotide.

All other parameters for this analysis have initial default values unless the user specifies them with the following lines:

N_min_5mer = <a number, default: 1>

- The minimum number for testing the number of signatures

N_max_5mer = <a number, default: 10>

- The maximum number for testing the number of signatures

CPU_5mer = <a number, default: 30>

- The number of allocated CPU cores for this analysis

nmf_iters_5mer = <a number, default: 1e4>

- Number of iterations for NMF algorithm in each epoch

nmf_conv_5mer = <a number, default: 1e-5>

- Convergence threshold for stopping NMF iterations

nmf_max_5mer = <a number, default: 5e5>

- Maximum number of iterations for NMF algorithm

boot_iters_5mer = <a number, default: 30 or number of available CPUs>

- Number of bootstrap iterations performed in each epoch

boot_conv_5mer = <a number, default: 0.01>

- The convergence threshold for stopping bootstrap iterations

boot_max_5mer = <a number, default: 600>

- Maximum number of bootstrap iterations

The results of this analysis are stored in the output directory in a folder named "infered_5mer_signatures".

5.3. Cluster samples based on contribution of 3-mer signatures

In this analysis, it is assumed that the 3-mer mutational signatures for the samples are already inferred in a previous run of CANCERSIGN or the analysis for inferring the signatures are enabled in the current configurations. To enable this analysis, write the following line in the configuration file:

```
cluster_samples_based_on_3mer_signatures = yes
```

There is not any additional parameter required for this analysis. The contributions of 3mer mutational signatures to the mutational profiles of samples are used as the bases for clustering the samples. The results of this analysis are stored in the output directory in a folder named "cluster_samples_based_on_3mer_signatures".

5.4. Cluster samples based on contribution of 5-mer signatures

In this analysis, it is assumed that the 5-mer mutational signatures for the samples are already inferred in a previous run of CANCERSIGN or the analysis for inferring the signatures are enabled in the current configurations. To enable this analysis, write the following line in the configuration file:

```
cluster_samples_based_on_5mer_signatures = yes
```

There is not any additional parameter required for this analysis. The contributions of 5mer mutational signatures to the mutational profiles of samples are used as the bases for clustering the samples. The results of this analysis are stored in the output directory in a folder named “cluster_samples_based_on_5mer_signatures”.

5.5. Cluster samples based on mutation counts in 3-mer motifs

To enable this analysis, write the following line in the configuration file:

```
cluster_samples_based_on_3mer_motifs = yes
```

The only parameter that the user must provide for this analysis is the desired set of 3-mer motifs. Based on this parameter, the counts of mutations in the specified motifs are used as the bases for clustering the samples. This parameter is specified as follows:

```
selected_3mer_motifs_for_clustering = <motif-1>, . . ., <motif-n>
```

where <motif-i> is a selected 3mer motif in a standard format. An example of this standard format is “**G[C>T]A**” which means C>T mutation with G as left flanking nucleotide and A as the right flanking nucleotide.

5.6. Cluster samples based on mutation counts in 6-mer motifs

To enable this analysis, write the following line in the configuration file:

```
cluster_samples_based_on_5mer_motifs = yes
```

The only parameter that the user must provide for this analysis is the desired set of 5-mer motifs. Based on this parameter, the counts of mutations in the specified motifs are used as the bases for clustering the samples. This parameter is specified as follows:

```
selected_5mer_motifs_for_clustering = <motif-1>, . . ., <motif-n>
```

where <motif-i> is a selected 5mer motif in a standard format. An example of this standard format is “**GC[T>A]AA**” which means T>A mutation with GC as left flanking dinucleotide and AA as the right flanking dinucleotide.
